# Targeted delivery of diverse biomolecules with engineered bacterial nanosyringes

**DOI:** 10.1038/s41587-025-02774-x

**Published:** 2025-08-12

**Authors:** Joseph Kreitz, Victoria Yang, Mirco J. Friedrich, Julie Pham, Rhiannon K. Macrae, Feng Zhang

**Affiliations:** 1 Howard Hughes Medical Institute, Cambridge, MA USA; 2 Yang Tan Collective, Cambridge, MA USA; 3 McGovern Institute for Brain Research at MIT, Cambridge, MA USA; 4 Broad Institute of MIT and Harvard, Cambridge, MA USA; 5 Department of Brain and Cognitive Science, Massachusetts Institute of Technology, Cambridge, MA USA; 6 Department of Biological Engineering, Massachusetts Institute of Technology, Cambridge, MA USA; 7 Department of Stem Cell and Regenerative Biology, Harvard University, Cambridge, MA USA

**Keywords:** Molecular biology, Biotechnology

## Abstract

The *Photorhabdus* virulence cassette is a microbial nanosyringe that can be engineered to deliver protein cargos into human cells. Here we further modify this system to incorporate exogenous cargos and targeting moieties in vitro. We show that this method, termed SPEAR, enables loading of different types of cargo (including folded ribonucleoproteins and single-stranded DNA) and targeting of defined cell types both in vitro and in vivo.

## Abstract (web summary)

Ribonucleoproteins, nucleic acids and proteins are delivered into target cell types with nanosyringes.

## Main

**Fig. 1:**
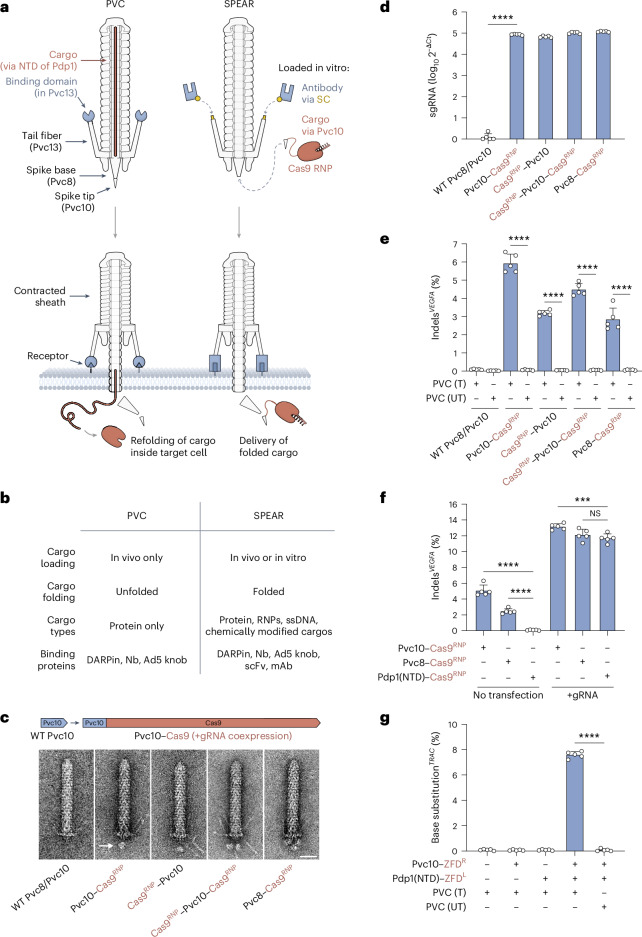
SPEAR is a modular system for cargo loading and retargeting with PVCs. **a**,**b**, SPEAR harnesses two attachment points to enable modular in vitro cargo loading (via self-assembly of Pvc10 onto Pvc8) and retargeting (via conjugation of antibodies to Pvc13). **c**, A cargo domain (Cas9) fused to the PVC spike (Pvc8) or spike tip (Pvc10) can be visualized loading onto PVCs. A novel density (designated with an arrow) can be observed at the base of the particle; scale bar, 25 nm. **d**, RT–qPCR analysis of the complexes from **c** indicates that Pvc8/Pvc10–Cas9 fusions can load onto PVCs as RNPs. **e**, PVCs can deliver Cas9 RNPs into HEK293FT cells. Cas9 activity required proper PVC targeting; T, targeted (Pvc13–Ad5 knob); UT, untargeted (truncated Pvc13). **f**, Comparison of spike-based cargo loading (this study) to tube-based (internal) cargo loading^13,21^. Cas9 loaded into the PVC tube (via N-terminal fusion of the Pdp1 N-terminal domain (NTD)) was unable to co-deliver sgRNA, while Pvc8/Pvc10–Cas9 yielded transfection-independent gene editing. **g**, Spike-based cargo loading can be combined with tube-based cargo loading to enable delivery of a two-component base editor (ZFD) with a single PVC design. All values in **d**–**g** are shown as mean ± s.d. with *n* = 5 biological replicates. Statistical significance was computed using one-way analysis of variance (ANOVA) with a Bonferroni post hoc test; ****P < 0.0001; ***P < 0.001; NS, not significant. Source data

Contractile injection systems (CISs) encompass a broad class of syringe-like protein complexes that enable prokaryotes and their viruses to inject effector molecules into nearby cells^1–12^. We previously reprogrammed an extracellular CIS (the *Photorhabdus* virulence cassette (PVC)) to deliver proteins into human cells^13^, highlighting this system’s potential for use as a therapeutic delivery technology. Here, we engineer PVCs to extend their utility, enabling delivery of different types of cargo and compatibility with a wider range of cell-type-specific targeting proteins, leading to a system termed spike engineering and retargeting (SPEAR; Fig. 1a,b).

**Extended Data Fig. 1:**
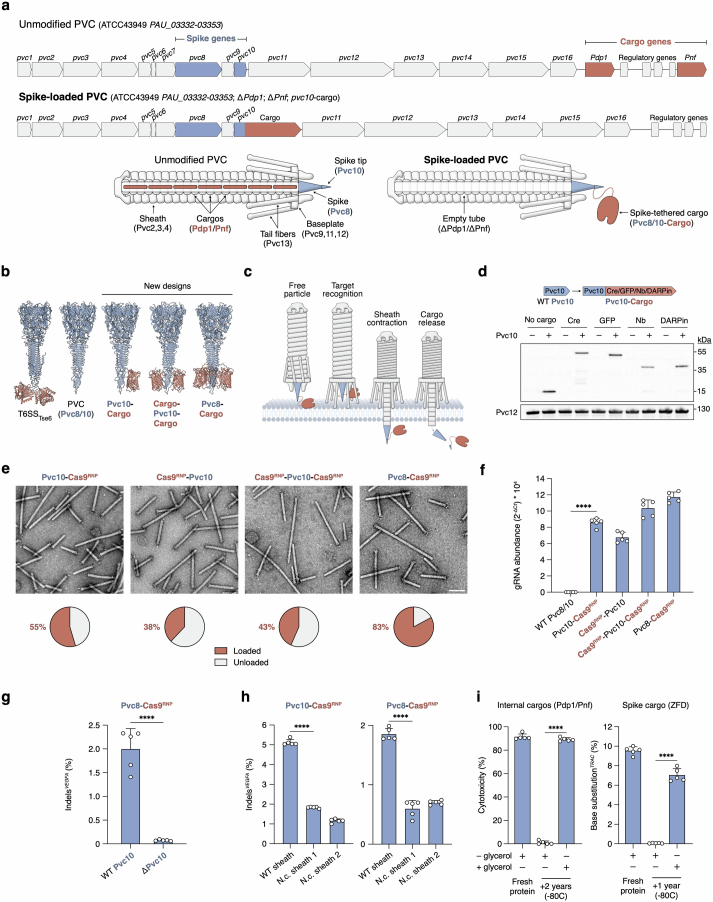
Design strategy and validation of Pvc8/Pvc10-mediated cargo loading. **a**, The wildtype PVCpnf locus—consisting of 2 spike genes (the spike (*pvc8*) and spike tip (*pvc10*), in blue) and 2 cargo genes (*Pdp1* and *Pnf*, in orange)—was redesigned to generate PVCs which load cargo via the spike complex. *Pdp1* and *Pnf* were deleted, and novel cargo domains were fused to either *pvc8* or *pvc10*. **b**, AlphaFold3 predicted structures of engineered Pvc8/Pvc10-cargo fusions share similarity with similar complexes from T6SSs. The spike-cargo effector from T6SS_Tse6_ (PDB: 4MTK/1TU1^14^) was used as a reference. GFP was used as the cargo domain in AlphaFold queries for novel spike-cargo designs. Note that Pvc8 assembles as a trimer; thus, three cargo domains are present in Pvc8-cargo designs. **c**, Proposed mechanism of spike-mediated cargo delivery in human cells. PVCs harboring cargos fused to the spike complex (Pvc8/Pvc10) recognize target cells via the tail fiber (Pvc13), initiating a series of conformational changes which result in translocation of the spike—along with cargo domains tethered to the spike—across the membrane. **d**, PVCs can load exogenous cargos via Pvc10. Four arbitrary proteins (Cre, GFP, a nanobody (Nb), and a DARPin) were fused to the C-terminus of the spike tip (Pvc10), and loading was assessed using immunoblot on purified PVC complexes. Novel domains were only present in purified PVC samples when fused to Pvc10, indicating Pvc10 mediated loading of these domains onto the PVC complex. Baseplate (Pvc12) served as a loading control. **e**, Characterization of loading efficiency for novel spike-cargo designs. PVCs loaded with Cas9 were imaged with TEM (as in Fig. 1c) and the proportion of loaded particles were quantified (*n* = 250 for each design). Ambiguous particles were counted as unloaded. Zoomed-out images provided as context for the images in Fig. 1c. Scale bar, 100 nm. **f**, The RT–qPCR analysis from Fig. 1d plotted on a linear scale reveals differences in gRNA loading consistent with the observed variability of Cas9 loading from (**e**). Compared to the N-terminal Pvc10 fusion (Cas9-Pvc10), the C-terminal Pvc10 fusion (Pvc10-Cas9)—which also loads RNPs more efficiently—correspondingly loads more gRNA. The dual-Cas9 fusion (Cas9-Pvc10-Cas9) and Pvc8 fusion (Pvc8-Cas9) load the most gRNA, likely due to these designs harboring multiple Cas9 monomers per loaded PVC particle. **g**, Pvc8-mediated delivery of Cas9 requires the presence of Pvc10. Δ*pvc10* PVCs harboring Pvc8-Cas9 failed to produce indels in HEK293FT cells, indicating a functional spike complex is necessary for delivery of Cas9 in these particles. **h**, PVC-mediated delivery is dependent on sheath contraction. PVC-mediated delivery of Cas9 RNPs (as in Fig. 1e, f) was inhibited with PVCs harboring 5aa insertions in the sheath (Pvc2) previously shown to inhibit sheath contraction in other CISs^32,33^. See Supplementary Tables 4 and 5 for amino acid sequences of novel mutants. N.c., non-contractile. **i**, Longterm storage of PVCs. Tube-loaded PVCs (loaded with endogenous toxins Pdp1 and Pnf^13^; left) or spike-loaded PVCs (loaded with ZFDs, as in Fig. 1g; right) were frozen at -80 °C for 23 months and 12 months, respectively, and were subsequently incubated with HEK293FT cells to determine the effect of storage on cytotoxicity or base editing efficiency, respectively. Successful storage required the presence of 5% glycerol (v/v). All values (**f**–**i**) are mean ± s.d. with *n* = 5 biological replicates. Statistical significance was computed using one-way ANOVA with Bonferroni post hoc test (**f**, **h**, **i**) or two-sided Student’s *t*-test (**g**); ****P < 0.0001. Source data

In our first-generation PVC design, we used the injection system’s native cargo loading mechanism, which is limited to proteins. To accommodate different cargos, we took inspiration from a related family of CISs (type VI secretion systems (T6SSs)), which can fuse cargo domains to the spike at the base of the complex^14–17^. Given the structural similarity between the T6SS spike complex (VgrG/PAAR) and the PVC spike complex (Pvc8/Pvc10)^3,14,18^, and the fact that T6SSs have been successfully engineered to load exogenous cargos via the spike^19,20^, we imagined implementing an analogous spike-mediated cargo loading strategy in PVCs (Extended Data Fig. 1a–c). After deleting endogenous cargos, we fused a heterologous cargo domain (Cas9) to the spike (Pvc8) or spike tip (Pvc10) in an arrangement similar to that seen in T6SSs and analyzed the resulting purified PVCs with negative-stain transmission electron microscopy (TEM). We identified nascent densities at the base of each PVC particle likely corresponding to loaded Cas9 (Fig. 1c and Extended Data Fig. 1d,e). Two separate densities were observed with a Pvc10 design carrying two Cas9 domains (Cas9–Pvc10–Cas9), and three were observed when Cas9 was fused to the trimeric Pvc8 (Pvc8–Cas9), confirming that these densities likely corresponded to Cas9 cargos loaded onto the spike complex.

Although we previously demonstrated that Cas9 could be loaded into PVCs internally (via the tube)^13^, we were unable to load Cas9 in the complex with single guide RNA (sgRNA), likely because cargos are unfolded during loading into the tube^21^. However, our TEM results in Fig. 1c suggested that cargos loaded onto the spike complex remain folded; therefore, we sought to test if we could use this method to load and deliver Cas9 ribonucleoproteins (RNPs). We found that PVCs harboring Pvc8/Pvc10–Cas9 fusions, when coexpressed with sgRNA, purified with an approximately five-log enrichment of sgRNA over PVCs harboring unmodified spike complexes (Fig. 1d and Extended Data Fig. 1f). Furthermore, when these PVCs (carrying an Ad5 knob domain in Pvc13 to allow for targeting of human cells^13^, used in all live-cell experiments in this work unless specified otherwise) were added to HEK293FT cells, we observed on-target insertions and deletions (indels) without co-transfection of sgRNA (Fig. 1e,f and Extended Data Fig. 1g,h), indicating that Cas9 RNPs loaded onto the PVC spike are also functionally delivered. Finally, we also found that spike-based cargo loading can be combined with tube-based cargo loading, enabling the delivery of both halves of a two-component base editing system (the zinc finger deaminase (ZFD)^22^) by a single PVC design (Fig. 1g and Extended Data Fig. 1i).

**Fig. 2:**
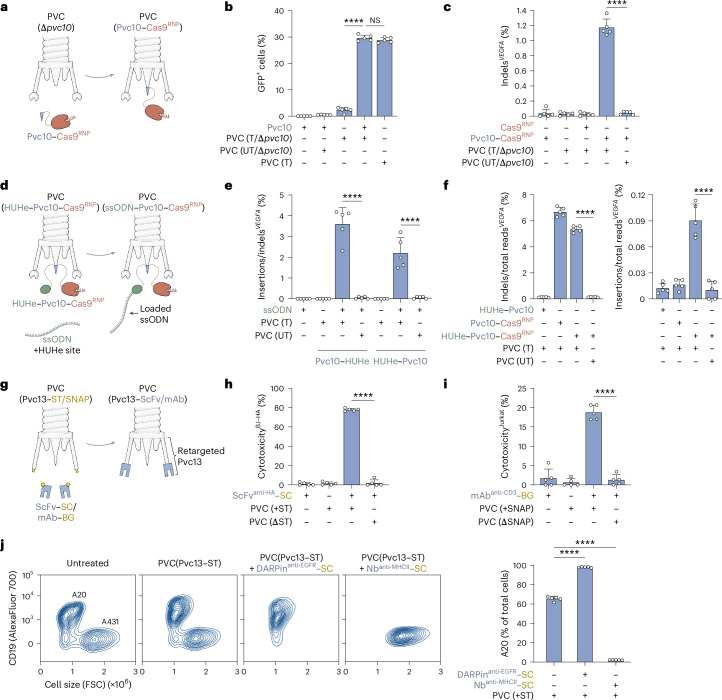
PVCs can be loaded and retargeted in vitro with SPEAR. **a**, Proposed model of in vitro cargo loading via Pvc10. Pvc10–cargo fusions can likely load onto PVCs via an interaction between Pvc10 and the untipped spike (Pvc8). **b**, Incubation with separately purified Pvc10 protein in vitro rescues the activity of Δ*pvc10* PVCs (loaded internally with Cre^13^) in HEK293FT cells co-transfected with *loxP*–GFP. Activity of Δ*pvc10* PVCs complemented with Pvc10 in vitro approached that of unmutated PVCs lacking the *pvc10* deletion. **c**, Δ*pvc10* PVCs incubated with Cas9 RNPs tethered to Pvc10 (Pvc10–Cas9^RNP^) in vitro produce on-target indels in HEK293FT cells. **d**, Proposed model of in vitro DNA loading via Pvc10. PVCs harboring an HUHe tag in Pvc10 are incubated with ssODNs containing an HUHe-specific recognition sequence, allowing for covalent conjugation of ssODNs to Pvc10, which enables spike-mediated translocation of ssDNA. **e**, PVCs loaded with ssODN HDR templates in vitro (as described in **d**) produce targeted 6-bp DNA insertions in HEK293FT cells following Cas9 genomic cleavage (conferred via pretransfection of a Cas9/sgRNA plasmid). **f**, PVCs can deliver DNA, RNA and protein simultaneously. PVCs loaded with Cas9 RNPs (as in Fig. 1e) and ssODN HDR templates (as in **e**) via a single multidomain Pvc10 design (HUHe–Pvc10–Cas9^RNP^) yield both indels and DNA insertions in untransfected HEK293FT cells. **g**, Proposed model of in vitro retargeting of PVCs using self-conjugating protein tags. PVCs containing ST or SNAP in the distal domain of the targeting element (Pvc13) are covalently conjugated to antibodies (scFvs or mAbs) labeled with SC or BG, altering the binding specificity of Pvc13. **h**,**i**, Endogenous toxin-loaded PVCs conjugated with an anti-HA tag single-chain antibody (scFv^anti-HA^) via ST/SC (**h**) or an mAb to CD3 (mAb^anti-CD3^) via SNAP/BG (**i**) kill human cells displaying the cognate binding partners for these antibodies (BJ–HA and Jurkat cells, respectively). **j**, PVCs retargeted in vitro are highly target specific, enabling programmable population editing. Toxin-loaded PVCs carrying Pvc13–ST were retargeted in vitro (as in **g**–**i**) with either an SC-labeled anti-EGFR DARPin (DARPin^anti-EGFR^–SC) or an SC-labeled anti-mouse MHC class II Nb (Nb^anti-MHCII^–SC) and were subsequently added to a mixed population of EGFR^+^ cells (A431) and MHC class II^+^ cells (A20). Retargeted PVCs selectively inhibited only one cell type within the mixed population. All values (**b**, **c**, **e**, **f** and **h**–**j**) are shown as mean ± s.d. with *n* = 5 biological replicates. Statistical significance was computed using one-way ANOVA with a Bonferroni post hoc test; ****P < 0.0001; NS, not significant. Source data

**Extended Data Fig. 2:**
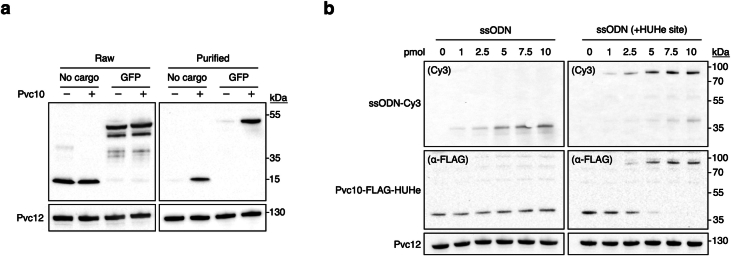
Biochemical analysis of *in vitro*-loaded PVCs. **a**, Pvc10-cargo fusion proteins self-associate with Δ*pvc10* PVCs *in vitro*. Pvc10 alone or with a cargo (GFP) were incubated with PVCs lacking Pvc10, purified using ultracentrifugation, and visualized with an immunoblot. Only intact Pvc10 or Pvc10-GFP successfully purified with the PVC complex after the complementation reaction; –Pvc10 variants of both (harboring a scrambled Pvc10 sequence) failed to purify. All proteins were tagged with FLAG; bands were visualized with anti-FLAG immunoblot. Baseplate (Pvc12) served as a loading control. **b**, ssODNs can be loaded onto the PVC spike tip *in vitro*. ssODNs (labeled with Cy3) were titrated against PVCs containing Pvc10-HUHe (tagged with FLAG), and protein-DNA conjugation was assessed using a dual Cy3/FLAG blot. Only ssODNs containing the 13-nt HUHe recognition sequence produced an increase in molecular weight in both the DNA and protein bands, indicating these species became covalently linked. Source data

In addition to delivering folded proteins and RNPs, Pvc10-based cargo loading may offer another advantage: because Pvc10 is an external component of the PVC and is not required for assembly of the full complex^3^, it might be able to self-assemble onto PVCs lacking Pvc10 in vitro, enabling loading of a wide variety of synthetic cargos (for instance, chemically modified cargos) that might be difficult to load in vivo (Fig. 2a). We found that separately purified Pvc10 protein readily assembles onto Δ*pvc10* PVCs in vitro, fully rescuing the activity of these particles (which were loaded with Cre internally^13^) against HEK293FT cells harboring a Cre reporter (*loxP*–green fluorescent protein (*loxP–*GFP); Fig. 2b). Furthermore, when Δ*pvc10* PVCs (lacking internal cargos) were incubated with Pvc10–Cas9 RNPs and added to HEK293FT cells, we observed on-target indels (Fig. 2c and Extended Data Fig. 2a), indicating the feasibility of this in vitro cargo loading approach.

We next sought to harness Pvc10 to load an entirely different category of cargos: single-stranded DNA (ssDNA; Fig. 2d). We fused an HUH endonuclease (HUHe) domain^23,24^ to Pvc10 and incubated the resulting Pvc10–HUHe PVCs with ~100-nt ssDNA oligonucleotides (ssODNs) containing both an HUHe-specific recognition sequence and a homology-directed repair (HDR) template designed to yield a 6-bp insertion in the human *VEGFA* locus (Extended Data Fig. 2b). After adding the resulting DNA-loaded PVCs to HEK293FT cells pretransfected with a Cas9/sgRNA plasmid, we observed the intended insertions at this locus (Fig. 2e). In addition, PVCs loaded with both Cas9 RNPs (via C-terminal fusion of Cas9 to Pvc10, as in Fig. 1c–f) and HUHe–ssODNs (via N-terminal fusion of HUHe to Pvc10 followed by in vitro conjugation of ssODNs, as in Fig. 2e) produced both Cas9-mediated indels and ssODN-mediated DNA insertions in untransfected HEK293FT cells (Fig. 2f), indicating that PVCs can deliver Cas9 RNPs and ssDNA HDR templates simultaneously via a single multidomain Pvc10 fusion.

**Extended Data Fig. 3:**
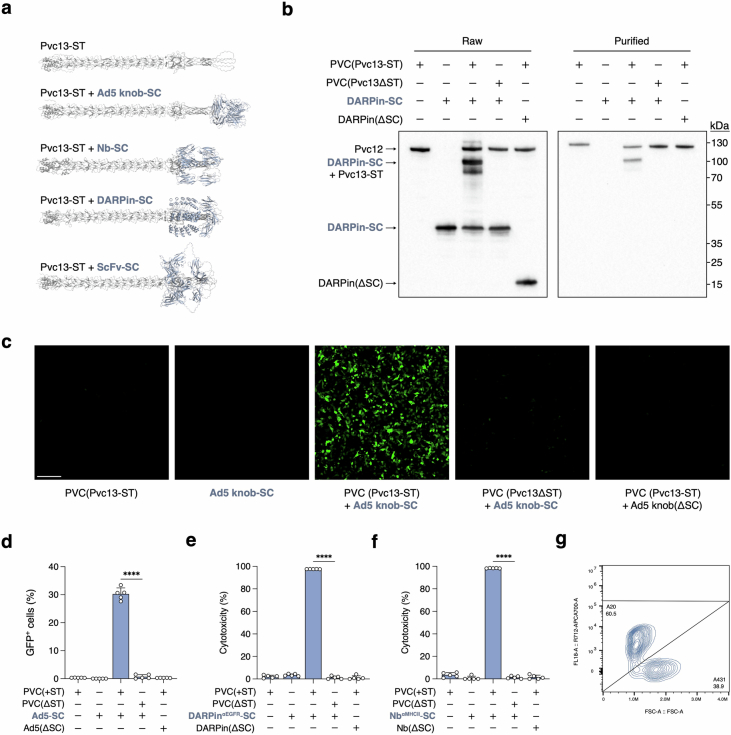
Biochemical and functional verification of *in vitro* retargeting. **a**, AlphaFold3 predicted structures of Pvc13-ST (grey) docked with various binding domain-SC designs (blue). Note that Pvc13 forms a trimer; therefore, three Pvc13-ST monomers and three binding domain-SC monomers were queried for each design. **b**, Pvc13 can be covalently linked with a novel binding domain using ST/SC. A DARPin fused to SC (DARPin-SC; tagged with FLAG) was incubated with PVCs harboring Pvc13-ST, and assembly of the DARPin/Pvc13 complex was assessed using anti-FLAG immunoblot. Assembly required both ST and SC in Pvc13 and the DARPin, respectively. Additionally, only the assembled DARPin-Pvc13 conjugate was retained in purified PVC fractions, indicating it was physically associated with the PVC complex. **c**, **d**, PVCs retargeted *in vitro* are active in human cells. PVCs loaded with Cre via Pdp1 (NTD) were retargeted with Ad5 knob domains *in vitro* (as was done with a DARPin in (**b**)) and exposed to HEK293FT cells co-transfected with a Cre reporter plasmid (*loxP*-GFP). Only particles resulting from an *in vitro* complementation reaction involving both ST and SC produced GFP activity. Images in (**c**); replicate data in (**d**). Scale bar, 200 μm. **e**, **f**, Individual validation of the retargeting moieties used in Fig. 2j. In addition to the Ad5 knob-SC used in (**c**, **d**), the anti-EGFR DARPin E01 (DARPin^αEGFR^-SC) (**e**) enabled PVC activity in EGFR+ cells (A431) and an anti-mouse MHC-II nanobody (Nb^αMHCII^-SC) (**f**) enabled PVC activity in MHC-II+ cells (A20). In these experiments, we used Pvc13-ST PVCs loaded with toxins; PVC-mediated delivery was detected as cytotoxicity. **g**, Flow cytometry gating scheme for the co-culture experiment in Fig. 2j. All values (**d**–**f**) are mean ± s.d. with *n* = 5 biological replicates. Statistical significance was computed using one-way ANOVA with Bonferroni post hoc test; ****P < 0.0001. Source data

To further extend the utility of PVCs for delivery, we sought to expand the breadth of cell types targetable by this system. Previously, we showed that small binding proteins, such as DARPins and nanobodies (Nbs), could be genetically inserted into the targeting element (Pvc13); however, larger targeting proteins (such as single-chain variable fragments (scFvs) and monoclonal antibodies (mAbs)) were incompatible with this strategy. To overcome this limitation, we inserted bioconjugation tags (SpyTag (ST) or SNAP-tag (SNAP)) into the distal binding domain of Pvc13, enabling us to covalently conjugate Pvc13 to full-sized antibodies (scFvs and mAbs) containing SpyCatcher (SC)^25,26^ or benzylguanine (BG)^27^ in vitro (Fig. 2g and Extended Data Fig. 3a,b). When PVCs harboring Pvc13–ST or Pvc13–SNAP were incubated with an anti-HA tag scFv fused to SC (scFv^anti-HA^–SC) or an mAb to CD3 labeled with BG (mAb^anti-CD3^–BG), respectively, we observed robust delivery activity in human cells displaying appropriate binding partners for these antibodies (BJ stably displaying HA tag (BJ–HA) or Jurkat cells, respectively; Fig. 2h,i and Extended Data Fig. 3c,d). Because the majority of publicly available binding proteins specific for human cell surface receptors are mAbs and scFvs, this result greatly expands the cell types and tissues targetable by PVCs.

**Extended Data Fig. 4:**
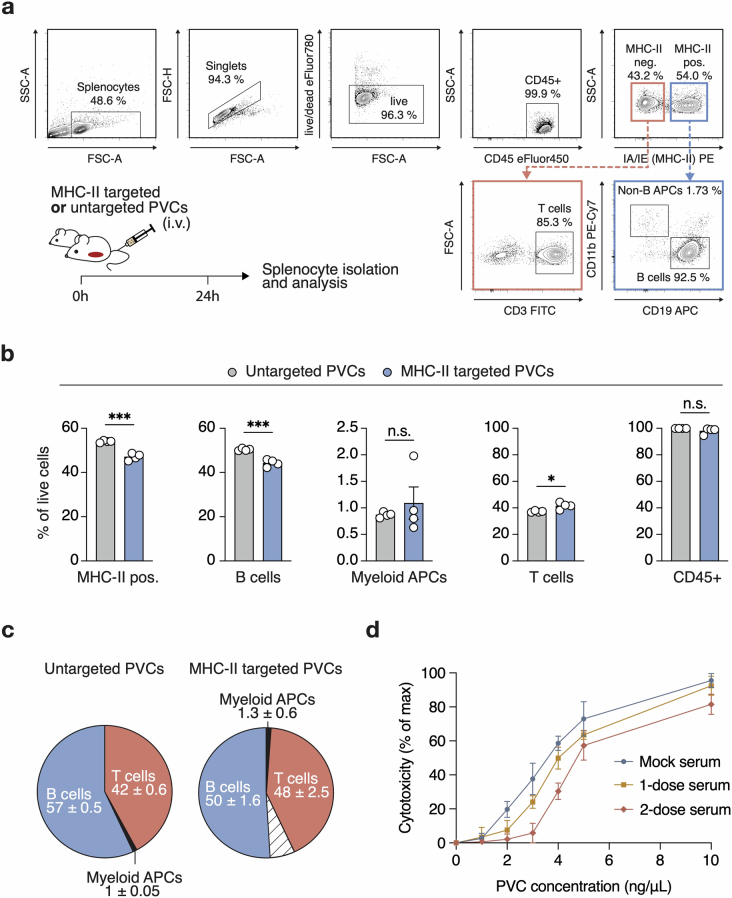
Validation of *in vitro*-retargeted PVCs *in vivo.* **a**, Schematic and flow cytometry workflow for *in vivo* studies of reprogrammed PVCs. Endogenous toxin-loaded PVCs retargeted against mouse MHC-II via ST/SC-based *in vitro* retargeting (as in Fig. 2j) were injected into mice (i.v.) and splenocytes were subsequently analyzed for targeted depletion of MHC-II positive cells. **b**, MHC-II-targeting PVCs selectively inhibit MHC-II positive cells *in vivo*. PVCs injected into mice as in (**a**) produced specific cytotoxicity against the MHC-II+ cell population, including B cells, without significant reduction in the total cell population (CD45+) or depletion of off-target populations (myeloid APCs and T cells). **c**, PVC injections alter the cellular composition of the mouse spleen. Systemic treatment with MHC-II-targeting PVCs (**a**, **b**) reduced the total B cell population in the spleen by about 7% after 24 h. **d**, PVC injections result in minor neutralizing immunity. Serum from mice challenged with a mock injection (PBS), one PVC injection, or two PVC injections (at *t* = 0 and 7 days) were extracted at *t* = 14 days and incubated with equivalent MHC-II targeting PVCs to determine the effect of serum on PVC activity in MHC-II positive cells (A20). PVCs incubated with serum from mice challenged with two PVC doses exhibited a roughly 30% decrease in potency (IC50) compared to PVCs incubated with mock serum. All values are mean ± s.d. with n = 4 biological replicates. Statistical significance was computed using two-sided Student’s *t*-test; ****P < 0.0001; ***P < 0.001; *P < 0.05; n.s., not significant. Source data

We next asked whether PVCs retargeted in vitro retain the high level of target specificity observed previously with retargeted PVCs^13^. Using the ST/SC retargeting method, we conjugated toxin-loaded PVCs with either of two different binding domains, the anti-mouse MHC class II Nb VHH7 (Nb^anti-MHCII^)^28^ or the anti-human EGFR DARPin E01 (DARPin^anti-EGFR^)^29^, and applied the resulting retargeted PVCs to an approximately 1:1 coculture of cells expressing either mouse MHC class II (A20) or human EGFR (A431). We found that each PVC treatment selectively depleted only a single subset of cells within the mixed population: PVCs conjugated with Nb^anti-MHCII^ selectively inhibited A20 cells, and PVCs functionalized with DARPin^anti-EGFR^ selectively inhibited A431 cells (Fig. 2j and Extended Data Fig. 3e–g). In addition, after systemic administration of similar anti-mouse MHC class II-targeting PVCs (conjugated with Nb^anti-MHCII^) into mice, we observed a roughly 7% bulk depletion of MHC class II^+^ cells in the mouse spleen without significant impact on off-target cell populations (Extended Data Fig. 4a–c) and with only minor neutralizing immunity (Extended Data Fig. 4d), indicating that PVCs functionalized in vitro can be leveraged to specifically target defined cell types in vivo. This could be useful for delivery applications targeting circulating immune cells; for instance, reprogramming B cells for sustained therapeutic production^30^.

**Extended Data Fig. 5:**
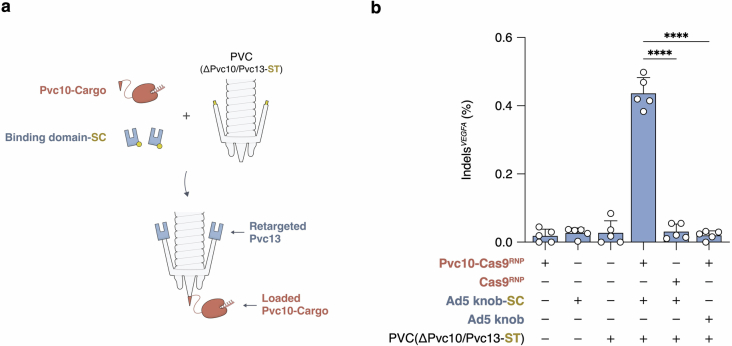
SPEAR enables modular cargo loading and retargeting in a single-pot reaction. **a**, Proposed model of dual-functionalization of PVCs via spike engineering and retargeting (SPEAR). Δ*pvc10* PVCs harboring Pvc13-ST were incubated with separately purified cargo (as in Fig. 2a–c) and targeting protein (as in Fig. 2g–i), yielding PVC complexes programmed to deliver defined molecular cargos into defined target cells. **b**, PVCs can be both loaded and retargeted *in vitro*. Indels were observed only when the cargo (Cas9 RNPs) contained Pvc10 and when the targeting protein (Ad5 knob) contained SC, indicating two separate functionalization steps occurred. All values are mean ± s.d. with *n* = 5 biological replicates. Statistical significance was computed using one-way ANOVA with Bonferroni post hoc test; ****P < 0.0001. Source data

Finally, we tested whether Pvc8/Pvc10-based in vitro cargo loading (Fig. 2a–f) and ST/SC-based in vitro retargeting (Fig. 2g–j) can be combined into a single in vitro complementation reaction (Extended Data Fig. 5a). We found that when PVCs lacking both a cargo and a targeting moiety (Δ*pvc10*/Pvc13–ST) were incubated with separately purified Pvc10–Cas9^RNP^ and Ad5 knob–SC, the resulting reprogrammed PVCs produced on-target edits in HEK293FT cells (Extended Data Fig. 5b), indicating that these particles were equipped with both a cargo and a targeting moiety in vitro. Overall, this study demonstrates that PVCs can be modularly functionalized with a variety of cargos (including protein, RNPs, ssDNA and multidomain cargos; Figs. 1c–g and 2a–f) and cell-type-specific binding proteins (including scFvs and mAbs; Fig. 2g–i and Extended Data Fig. 3), enabling target-specific delivery both in artificial mixed populations (Fig. 2j) and within live mice (Extended Data Fig. 4b,c). Due to the PVC’s unique injection-based mechanism of action (Extended Data Fig. 1c), which is thought to operate independently of intrinsic cellular pathways for internalization or trafficking, this system is likely well suited for use in metabolically inactive or senescent cells or in nonhuman organisms that are difficult to target with existing tools (such as plants, fungi or prokaryotes). Together, these results establish SPEAR as a highly programmable chassis for biomolecular delivery with wide-ranging applications in biotechnology.

## Methods

### Plasmid construction

The wild-type PVCpnf locus was constructed previously^13^ and is publicly available as separate structural (*pvc1*–*pvc16*) and cargo/regulatory (*Pdp1*, *Pnf* and regulatory genes *PAU_RS16570–PAU_RS24015*) plasmids (Addgene, 198271 and 198272, respectively). Routine plasmid manipulations involved PCR amplification using KOD ONE polymerase (Sigma, KMM-101NV; 6-min extension time for structural plasmids and 2-min extension time for cargo/regulatory plasmids), assembly with Gibson Assembly Master Mix (New England Biolabs, E2611L) and either electroporation into EPI300 electrocompetent cells (Lucigen, EC300110; for structural plasmids) or transformation into chemically competent Stbl3 cells (Thermo Fisher, C737303; for cargo/regulatory plasmids). Novel designs were structurally validated with AlphaFold3 and visualized with PyMOL (2.5.2) and ChimeraX (1.8). A list of amino acid/DNA sequences for constructs used throughout this study can be found in Supplementary Tables 1–10.

### PVC expression and purification

PVC samples were obtained using a previous method^3,13^ with modifications. Briefly, one variant each of the PVC structural plasmid and cargo/regulatory plasmid were electroporated into EPI300 cells, after which colonies were inoculated into 5 ml of Terrific Broth (TB; US Biological, T2810) and shaken at 37 °C for 16 h. Starter cultures were then inoculated (at 1:100) in 500 ml of TB medium and shaken at 24 °C for an additional 48 h. Cultures were then spun for 25 min at 4,000*g*, resuspended in 22 ml of lysis buffer (25 mM Tris-HCl (pH 7.5; Thermo Fisher, 15567027), 140 mM NaCl (AmericanBio, AB01915), 3 mM KCl (Sigma-Aldrich, P9541), 5 mM MgCl_2_ (Sigma-Aldrich, M4880), 200 μg ml^−1^ lysozyme (Thermo Fisher, 89833), 50 μg ml^−1^ DNase I (Sigma-Aldrich, DN25), 0.5% Triton X-100 (Sigma-Aldrich, 93443) and 1× Protease Inhibitor Cocktail (MedChem Express, HY-K0010)) and subsequently shaken at 37 °C for 60 min to promote lysis. Lysates were then pelleted at 5,000*g* for 30 min at room temperature (RT) to remove bulk cell lysate. Supernatants were then spun in an ultracentrifuge at 120,000*g* for 1 h at 4 °C to pellet PVC protein complexes. Pellets were hydrated overnight at 4 °C in 1 ml of PBS (Life Technologies, 10010049), resuspended and spun at 16,000*g* for 15 min at 16 °C to remove residual solid lysate. Supernatants were then applied to 22 ml of cold PBS before repeating the ultracentrifuge spin (120,000*g* for 1 h) and resuspending in 100–500 µl of cold PBS (with the final volume depending on pellet size). Final resuspensions were again clarified at 16,000*g* for 15 min at 16 °C, and PVC yields were quantified by A280 measurement on a NanoDrop instrument (Thermo Fisher). For mouse experiments, endotoxin was then removed from the final PVC samples using a detergent-based method used previously^13^. All PVC samples containing spike–cargo fusions were used immediately; all other PVCs were stored at 4 °C for a maximum of 2 weeks before use. Long-term storage of PVCs was achieved by adding 5% (final concentration) glycerol to the PVC suspension and storing at –80 °C without flash-freezing.

### Affinity purification of spike–cargo fusions and binding domain–SC fusions

Several proteins used to functionalize PVCs in vitro (such as the Pvc10–Cas9^RNP^ protein used in Fig. 2c and all cargo/targeting proteins used in Extended Data Fig. 5b) were purified in isolation of PVCs using affinity chromatography. Plasmids carrying the desired open reading frames with N-terminal affinity tags (6×His-TwinStrep-Sumo) were transformed into Rosetta 2 (DE3) cells (Sigma-Aldrich, 71401M). Each colony was inoculated into 10 ml of TB medium and grown at 37 °C for 14–18 h before being inoculated into an additional 500 ml of TB. Cultures were grown at 37 °C until they reached an optical density at 600 nm of 0.6–0.8, induced with 1 mM isopropyl β-d-1-thiogalactopyranoside (GoldBio, I2481C) and subsequently allowed to grow at 18 °C for an additional 16–18 h. Cells were then collected via centrifugation at 4,000*g* for 30 min, resuspended in 15 ml of cold lysis buffer (50 mM Tris-HCl (pH 7.5; Thermo Fisher, 15567027), 280 mM NaCl (AmericanBio, AB01915), 3 mM KCl (Sigma-Aldrich, P9541), 5 mM MgCl_2_ (Sigma-Aldrich, M4880), 20 mM imidazole (VWR, IC102033), 1× cOmplete Protease Inhibitor Cocktail (Sigma-Aldrich, 11836170001) and 50 µg ml^−1^ DNase I (Sigma-Aldrich, DN25); 0.004 U µl^−1^ SUPERase-In RNase Inhibitor (Thermo Fisher, AM2694) if purifying RNPs) and passed twice through a Microfluidics M110P microfluidizer. Lysates were then clarified at 19,000*g* for 20 min at 4 °C, after which they were incubated with 2 ml of 50% Ni-NTA agarose (Qiagen, 30210; pre-equilibrated in lysis buffer) with shaking for 1 h at 4 °C. Following incubation, Ni-NTA beads were pelleted by centrifugation at 1,000*g* for 3 min at 4 °C, transferred to a column (Thermo Fisher, 29922), washed with 10 ml of cold lysis buffer, washed with 10 ml of cold wash buffer 1 (40 mM imidazole in PBS; Life Technologies, 10010049), washed with 10 ml of cold wash buffer 2 (60 mM imidazole in PBS) and finally eluted with 2 ml of cold elution buffer (250 mM imidazole in PBS) for 5 min. The elution was collected, suspended in excess cold PBS and concentrated using an Amicon Ultra centrifugal filter unit (molecular weight cutoff of 10 kDa or 50 kDa; Sigma-Aldrich, UFC9050). Purified protein yield was quantified by measuring A280 on a NanoDrop instrument (Thermo Fisher). For cargos containing sgRNA, 1 U µl^−1^ SUPERase-In was also added to prevent RNase activity. To remove the 6×His-TwinStrep tag, purified proteins were treated with Ulp1 protease (a gift from S. Vo, Broad Institute of MIT and Harvard) at 0.4 mg of Ulp1 per 1 mg of cargo at RT for 1.5 h.

### In vitro expression of spike–cargo fusions and binding domain–SC fusions

Several proteins used to functionalize PVCs in vitro (such as the Pvc10 protein in Fig. 2b, the anti-MHC class II/anti-EGFR proteins in Fig. 2j and the Ad5–SC protein in Extended Data Fig. 3c,d) were expressed using an in vitro transcription/translation (IVTT) system. The desired open reading frames were synthesized as gBlocks (IDT) containing 5′ and 3′ extensions required for expression in a T7-based IVTT system (5′ T7 promoter and ribosome binding site; 3′ T7 terminator) according to the manufacturer’s guidelines. gBlocks were enriched using PCR with NEBNext polymerase (New England Biolabs, M0541), and the resulting amplicons were purified using a PCR purification kit (Qiagen, 28104) and eluted in distilled water. One microgram of each purified amplicon was then spiked into PURExpress reactions (New England Biolabs, E6800; 50 µl final volume) and incubated at 37 °C for 3 h to promote expression of the desired protein. These reactions were then used as raw inputs for in vitro complementation reactions (see ‘In vitro cargo complementation’ and ‘In vitro retargeting’ below).

### In vitro cargo complementation

To complement PVCs with spike–cargo fusion proteins in vitro, 150 µg of PVCs was incubated with either 15 µg of affinity-purified spike–cargo protein in a 50-µl reaction (in PBS) or with 50 µl of a raw IVTT reaction containing a spike–cargo protein; these reactions were incubated for 1–2 h at 24 °C. For cargos containing Cas9 RNPs, 1 U µl^−1^ SUPERase-In (final concentration) was also added before the addition of cargo. Reactions were then diluted to 100 µl with PBS and purified with 100 µl of a 50% slurry (in PBS) of Capto Core 700 resin (Cytiva, 17548101). Reactions were incubated with resin for 30 min at RT with rotation, after which they were transferred to a filter plate (Harvard Apparatus, 74-5650) and eluted by centrifugation at 1,500*g* for 3 min. An amino acid sequence for an engineered Pvc10 construct designed to load Cas9 onto PVCs can be found in Supplementary Table 1.

### In vitro ssDNA complementation

To load ssDNA cargos onto PVCs in vitro, 150 µg of PVCs (containing Pvc10–HUHe) was mixed with 10 pmol of DNA (containing an HUHe recognition sequence: AAGTATTACCAGC; synthesized by Azenta) in a 50-µl reaction in HUH buffer (50 mM HEPES (pH 8), 50 mM NaCl, 1 mM MgCl_2_ and 1 mM MnCl_2_), which was incubated at 37 °C for 2 h to promote conjugation of HUHe to the DNA cargo. Protein–DNA conjugation reactions were then purified using Capto Core 700 pre-equilibrated with HUH buffer before DNA delivery experiments in live cells. An amino acid sequence for an engineered Pvc10 construct harboring HUHe, as well as a DNA sequence for a representative HUHe-specific ssDNA cargo, can be found in Supplementary Tables 6 and 7.

### In vitro retargeting

To alter PVC target specificity in vitro, 150 µg of PVCs was incubated with either 5 µg of affinity-purified SC/BG-labeled binding protein (purified as described in ‘Affinity purification of spike–cargo fusions and binding domain–SC fusions’ or provided by B. Lash, M. Segel and D. Strebinger, Broad Institute of MIT and Harvard) in a 50-µl reaction (in PBS) or with 50 µl of a raw IVTT reaction containing a binding domain–SC protein (see ‘In vitro expression of spike–cargo fusions and binding domain–SC fusions’); these reactions were incubated for 1 h at RT. Retargeted PVCs were then purified using Capto Core 700 (see ‘In vitro cargo complementation’) before experiments in live cells. Amino acid/DNA sequences for an ST-functionalized PVC tail fiber (Pvc13–ST), a binding domain–SC protein used to retarget Pvc13–ST and a DNA amplicon used to express a binding domain–SC protein via IVTT can be found in Supplementary Tables 8–10.

### Biochemical assessment of cargo loading

Loading of cargo domains onto Pvc8/Pvc10 was verified using immunoblots. Ten micrograms of purified PVCs (containing spike–cargo fusion proteins) was mixed with NuPAGE LDS Sample Buffer (Thermo Fisher, NP0008) and NuPAGE Sample Reducing Agent (Thermo Fisher, NP0009), both to a final concentration of 1×, and were subsequently boiled at 95 °C for 10 min. The denatured samples were then run on NuPAGE Bis-Tris 1–12% protein gels (Thermo Fisher, NP0321) for 20 min at 200 V in 1× MOPS buffer (Thermo Fisher, NP000102) and were blotted onto PVDF membranes using an iBlot 2 instrument (Thermo Fisher). Membranes were blocked for 1 h at RT in blocking buffer (5% BLOT-QuickBlocker (VWR, 786-011) in 1× TBS-T (Thermo Fisher, 28360)), stained for 1 h at RT with mouse anti-Flag (Sigma, F3165; diluted 1:1,000 in blocking buffer), washed three times with TBS-T (5 min each), stained for 30 min with anti-mouse IgG (Cell Signaling, 7076; diluted 1:1,000 in blocking buffer) and washed another three times with TBS-T (5 min each). Finally, membranes were incubated for 1 min with chemiluminescence substrate (Thermo Fisher, 32209) and imaged with a Bio-Rad ChemiDoc instrument. For ssDNA loading experiments, the Cy3 channel was also imaged alongside chemiluminescence. Pvc12 was used as a loading control for immunoblots; this protein was visualized via the addition of a C-terminal Flag tag.

### Electron microscopy

Loading of Cas9 onto the PVC spike complex was visualized using negative-stain TEM. Briefly, PVC samples were diluted to 100–500 ng µl^−1^ in PBS and applied to a glow-discharged 200-mesh carbon-coated copper TEM grid (VWR, 100489-722) for 60 s before removing excess liquid with a light-duty tissue wiper (VWR, 82003). Grids were then treated with clarified 2% uranyl formate stain (incubating with gentle agitation for 5 s, 5 s, 10 s, 20 s and 20 s on five separate 30-µl uranyl formate droplets positioned on Parafilm) and allowed to dry at RT. TEM imaging was performed using an FEI Tecnai (G2 Spirit TWIN) microscope at 120 kV equipped with a Gatan Orius SC1000B camera located in the MIT.nano Characterization Facilities at Massachusetts Institute of Technology.

### Quantification of sgRNA loading onto Pvc8/Pvc10–Cas9 complexes

To confirm the presence of sgRNA in PVCs loaded with Cas9 RNPs, we performed quantitative reverse transcription PCR (RT–qPCR). PVCs were standardized to a concentration of 1,000 ng µl^−1^ and denatured by mixing with NuPAGE Sample Reducing Agent (Thermo Fisher, NP0009; 1× final concentration) and boiling at 95 °C for 10 min. These denatured samples were then used to generate cDNA using ProtoScript II Reverse Transcriptase (New England Biolabs, M0368) and random primers (New England Biolabs, S1330), according to the manufacturer’s protocol. We then ran qPCR on the resulting cDNAs using Fast SYBR Green Master Mix (Thermo Fisher, 4385612) in a Bio-Rad CFX Opus 384 qPCR instrument. A list of RT–qPCR primers used during this study can be found in Supplementary Table 11.

### Cell culture

All cell lines were maintained in T75 flasks (Thermo Fisher, 156499) at 37 °C with 5% CO_2_ in either DMEM-GlutaMAX (Thermo Fisher, 10569044; for HEK293FT and A431 cells) or RPMI-GlutaMAX (Thermo Fisher, 61870127; for A549 and A20 cells). All media were supplemented with 10% fetal bovine serum (VWR, 97068-085) along with 10 µg ml^−1^ gentamicin (Sigma-Aldrich, G1397) and 1× penicillin–streptomycin (Thermo Fisher, 15140122) to prevent bacterial growth. A list of cell lines used in this study can be found in Supplementary Table 12.

### Live-cell experiments with PVCs

To assess the activity of PVCs in live cells, we first seeded target cells into clear-bottom, 96-well plates (VWR, 89091-012) and allowed them to grow to ~80% confluence. A total of 7.5 µg of purified PVCs (into 100 µl of cell culture medium) was then added to each well. For some experiments, improved efficiency was observed if this dose was spaced over 3 days (2.5 µg per day). For experiments involving co-transfection, DNA was transfected using GeneJuice Transfection Reagent (Sigma-Aldrich, 70967) either immediately after adding PVCs (for Cre reporter plasmids) or 6 h before adding PVCs (for Cas9/sgRNA or bdSENP1 plasmids; these transfections were followed by a medium exchange before adding PVCs). For ssODN delivery experiments, 1 µM (final concentration) Alt-R HDR enhancer V2 (IDT, 10007921) was also added alongside PVCs to promote HDR-mediated repair. For toxin delivery experiments, cytotoxicity was assessed using CellTiter-Glo 2.0 Cell Viability Assay (Promega, G9241) 24 h after adding PVCs; any wells exhibiting higher luminescence than the control well (PBS) were assigned a cytotoxicity value of 0% to avoid negative cytotoxicity. For assays involving Cre-driven GFP expression, cells were incubated for 3 days, imaged with a Leica DMi8 confocal microscope and analyzed by flow cytometry (see ‘Flow cytometry’). For gene editing experiments, cells were incubated for 4 days, genomic DNA was extracted with 50 µl of QuickExtract DNA Extraction Solution (Lucigen, QE09050), and indels/base substitutions were quantified by next-generation sequencing (see ‘Deep sequencing’). All numerical data from the PVC experiments were plotted with Prism (10.2.2), and figures were graphically assembled in Adobe Illustrator (27.1.1).

### Flow cytometry

Cells were first collected by incubation with 30 µl of TrypLE Express dissociation reagent (Thermo Fisher, 12604) for 5 min at RT, followed by resuspension with 50 µl of cold flow cytometry buffer (PBS supplemented with 2% EDTA (Life Technologies, 15575020) and 5% fetal bovine serum (VWR, 97068-085)). Samples were then run on a Beckman Coulter Cytoflex S flow cytometer, and analysis was performed using FlowJo (10.8.2).

### Specificity assay with mixed populations

To assess the target specificity of in vitro-retargeted PVCs (Fig. 2j), we instead used a modified protocol involving cocultures of two cell lines. Briefly, an approximately 1:1 mixture of A431 and A20 cells (1 × 10^6^ cells each) was seeded into 2 ml of RPMI-GlutaMAX (with supplements listed in ‘Cell culture’) in six-well, flat-bottom plates (VWR, 29442), 150 µg of in vitro-retargeted PVCs was added, and the cultures were allowed to grow for 48 h. The supernatants (containing suspension cells) were then collected and combined with adherent cells (collected in a similar manner as detailed in ‘Flow cytometry’), pelleted at 300*g* for 5 min, resuspended in 500 µl of fresh flow cytometry buffer to remove residual TrypLE reagent, immunostained with anti-mouse CD19–Alexa Fluor 700 (Thermo Fisher, 56-0193-82; 1:100 dilution) at RT for 30 min in the dark, washed with blank flow cytometry buffer three times and analyzed on a Beckman Coulter Cytoflex S flow cytometer. The flow cytometry gating scheme used during this experiment can be found in Extended Data Fig. 3g.

### Deep sequencing

We used deep sequencing to detect PVC-mediated delivery of gene editing systems (Cas9 and ZFDs). We first amplified the desired target region out of each genomic DNA extract using NEBNext (New England Biolabs, M0541) and subsequently barcoded the resulting amplicons with indexed Illumina P5 and P7 next-generation sequencing primers. Libraries were purified with a PCR purification kit (Qiagen, 28104), quantified on a NanoDrop instrument (Thermo Fisher) and sequenced on an Illumina MiSeq instrument. Indels/base substitutions were then quantified with Geneious Prime (2020.0.5). A list of primers used for deep sequencing can be found in Supplementary Table 13.

### In vivo B cell depletion assay

All experiments in mice conformed to guidelines established by the National Institutes of Health and were conducted under Institutional Animal Care and Use Committee protocol 0017-09-14-3, approved by the Broad Institute of MIT and Harvard. Animals were chosen randomly for treatment with either control or experimental conditions without blinding. Female C57BL/6J mice (aged 8–12 weeks) were obtained from the Jackson Laboratory (strain 000664). All mice were maintained on a 12-h light/12-h dark cycle with ad libitum access to food and water. A total of 100 µl per mouse at 1.2 µg µl^−1^ of either MHC class II-targeting or untargeted PVC in sterile 0.9% NaCl was injected intravenously. Animals were deeply anaesthetized 24 h after injection with CO_2_, and spleens of PVC-injected mice were extracted. Single-cell suspensions were generated by mashing through 100- and 70-µm cell strainers (Greiner One-Bio, 542000 and 542070) and repeated washing with PBS. Splenocyte samples were blocked with 1:50 TruStain FcX (anti-mouse CD16/CD32) reagent (BioLegend, 101320) before antibody staining for flow cytometry and stained with fixable viability dye eFluor780 (1:1,000 dilution; Thermo Fisher Scientific), anti-mouse CD45-eFluor450 (1:100 dilution; BioLegend), anti-mouse CD3-FITC (1:100 dilution; BioLegend), anti-mouse CD19-APC (1:100 dilution; BioLegend), anti-mouse CD11b-PE-Cy7 (1:100 dilution; BioLegend) and anti-mouse IA/IE (MHC class II)-PE (1:100 dilution; BioLegend) at 4 °C for 30 min. Flow cytometry was performed on a Beckman Coulter CytoFlex S device with standard laser and detector configuration, and data were analyzed using FlowJo 10.8.1 software.

### Serum neutralization assay

To assess whether mice mount a neutralizing immune response against PVCs following systemic injection, we used a serum neutralization assay. Briefly, we injected mice with either PBS (mock injection) or MHC class II-targeting PVCs (intravenously as described in ‘In vivo B cell depletion assay’), repeated the injections in one cohort at *t* = 7 days (to amplify the generation of neutralization antibodies), extracted serum for all cohorts at *t* = 14 days via submandibular bleed, incubated clarified serum with similar MHC class II-targeting PVCs (5% (vol/vol) serum and 15 µg of PVCs in each 100-µl reaction, diluted in PBS) for 1 h at RT and titrated the resulting PVC/serum mixtures on MHC class II^+^ cells (A20). Cytotoxicity was assessed as described in ‘Live-cell experiments with PVCs’.

### Statistics and reproducibility

All statistical analyses were performed in Prism (10.2.2). All quantitative data are presented as mean ± s.d. with *n* = 5 biological replicates per condition; biological replicates represent independent treatments in separate culture wells or mice. All images and blots are representative examples from *n* = 3 independent experiments. Statistical significance was computed using two-sided unpaired Student’s *t*-test or one-way ANOVA, followed by a Bonferroni post hoc test (to correct for multiple comparisons), as indicated in the figure legends.

### Reporting summary

Further information on research design is available in the Nature Portfolio Reporting Summary linked to this article.

## Online content

Any methods, additional references, Nature Portfolio reporting summaries, source data, extended data, supplementary information, acknowledgements, peer review information; details of author contributions and competing interests; and statements of data and code availability are available at 10.1038/s41587-025-02774-x.

## Supplementary information

Reporting Summary

Peer Review File

Supplementary Tables 1–13 Amino acid/DNA sequences of novel constructs, guide/primer sequences and cell line information.

## Source data

Source Data Fig. 1 Statistical source data.

Source Data Fig. 2 Statistical source data.

Source Data Extended Data Figs. 1–3 Unprocessed blot images.

Source Data Extended Data Fig. 1 Statistical source data.

Source Data Extended Data Fig. 3 Statistical source data.

Source Data Extended Data Fig. 4 Statistical source data.

Source Data Extended Data Fig. 5 Statistical source data.

## Data Availability

All plasmids used in this study are available on Addgene. Raw sequencing data are available from the Sequencing Read Archive under BioProject ID PRJNA1287998 (ref. ^31^). Source data are provided with this paper.
