# Supplementary material for: Targeted delivery of diverse biomolecules with engineered bacterial nanosyringes
Source: Nat Biotechnol. 2025 Aug 12;44(7):1121–5. doi: 10.1038/s41587-025-02774-x (PMC13368577; doi:10.1038/s41587-025-02774-x)
Supplement: Supplementary file 1 — Reporting Summary [file 41587_2025_2774_MOESM1_ESM.pdf]

nature portfolio

Corresponding author(s): Feng Zhang

Last updated by author(s): 7/08/2025

Reporting Summary

Nature Portfolio wishes to improve the reproducibility of the work that we publish. This form provides structure for consistency and transparency in reporting. For further information on Nature Portfolio policies, see our Editorial Policies and the Editorial Policy Checklist.

Statistics

For all statistical analyses, confirm that the following items are present in the figure legend, table legend, main text, or Methods section.

n/a	Confirmed	
☐	☒	The exact sample size (*n*) for each experimental group/condition, given as a discrete number and unit of measurement
☐	☒	A statement on whether measurements were taken from distinct samples or whether the same sample was measured repeatedly
☐	☒	The statistical test(s) used AND whether they are one- or two-sided<br>*Only common tests should be described solely by name; describe more complex techniques in the Methods section.*
☒	☐	A description of all covariates tested
☐	☒	A description of any assumptions or corrections, such as tests of normality and adjustment for multiple comparisons
☐	☒	A full description of the statistical parameters including central tendency (e.g. means) or other basic estimates (e.g. regression coefficient) AND variation (e.g. standard deviation) or associated estimates of uncertainty (e.g. confidence intervals)
☐	☒	For null hypothesis testing, the test statistic (e.g. *F*, *t*, *r*) with confidence intervals, effect sizes, degrees of freedom and *P* value noted<br>*Give P values as exact values whenever suitable.*
☒	☐	For Bayesian analysis, information on the choice of priors and Markov chain Monte Carlo settings
☒	☐	For hierarchical and complex designs, identification of the appropriate level for tests and full reporting of outcomes
☒	☐	Estimates of effect sizes (e.g. Cohen's *d*, Pearson's *r*), indicating how they were calculated

Our web collection on statistics for biologists contains articles on many of the points above.

Software and code

Policy information about availability of computer code

Data collection	We used AlphaFold3 (https://alphafoldserver.com/) to predict 3-dimensional protein structures.
Data analysis	We used Geneious Prime (2020.0.5) to quantify indels/base substitutions, PyMOL (2.5.2) and ChimeraX (1.8) to visualize protein structures, Prism (10.2.2) to generate data plots and run statistical tests, FlowJo (10.8.2) to analyze flow cytometry data, and Adobe Illustrator (27.1.1) to generate figures.

For manuscripts utilizing custom algorithms or software that are central to the research but not yet described in published literature, software must be made available to editors and reviewers. We strongly encourage code deposition in a community repository (e.g. GitHub). See the Nature Portfolio guidelines for submitting code & software for further information.

Data

Policy information about availability of data

All manuscripts must include a data availability statement. This statement should provide the following information, where applicable:

- Accession codes, unique identifiers, or web links for publicly available datasets
- A description of any restrictions on data availability
- For clinical datasets or third party data, please ensure that the statement adheres to our policy

All plasmids used in this study are available on Addgene. Raw sequencing data are available from the Sequencing Read Archive under BioProject ID PRJNA128799833. All other data (including uncropped immunoblot images, raw quantitative data, and statistical analyses) are provided in the Source Data.

Research involving human participants, their data, or biological material

Policy information about studies with human participants or human data. See also policy information about sex, gender (identity/presentation), and sexual orientation and race, ethnicity and racism.

Reporting on sex and gender	N/A - we did not use human research participants.
Reporting on race, ethnicity, or other socially relevant groupings	N/A - we did not use human research participants.
Population characteristics	N/A - we did not use human research participants.
Recruitment	N/A - we did not use human research participants.
Ethics oversight	N/A - we did not use human research participants.

Note that full information on the approval of the study protocol must also be provided in the manuscript.

Field-specific reporting

Please select the one below that is the best fit for your research. If you are not sure, read the appropriate sections before making your selection.

☒ Life sciences ☐ Behavioural & social sciences ☐ Ecological, evolutionary & environmental sciences

For a reference copy of the document with all sections, see nature.com/documents/nr-reporting-summary-flat.pdf

Life sciences study design

All studies must disclose on these points even when the disclosure is negative.

Sample size	Sample size (n) was set to 5 where possible. No a priori calculations of sample size were performed.
Data exclusions	No data were excluded from this study.
Replication	All replicates represent biological replicates (independent treatments in separate wells) with n = 5. All micrographs, gels, and blots are representative images from at least n = 3 independent experiments.
Randomization	Sample randomization was not applicable for cell culture experiments; all conditions received equivalent numbers of cells in separate wells.
Blinding	Blinding was not performed.

Reporting for specific materials, systems and methods

We require information from authors about some types of materials, experimental systems and methods used in many studies. Here, indicate whether each material, system or method listed is relevant to your study. If you are not sure if a list item applies to your research, read the appropriate section before selecting a response.

Materials & experimental systems

n/a	Involved in the study	
☐	☒	Antibodies
☐	☒	Eukaryotic cell lines
☒	☐	Palaeontology and archaeology
☐	☒	Animals and other organisms
☒	☐	Clinical data
☒	☐	Dual use research of concern
☒	☐	Plants

Methods

n/a	Involved in the study	
☒	☐	ChIP-seq
☐	☒	Flow cytometry
☒	☐	MRI-based neuroimaging

Antibodies

Antibodies used	All immunoblots used anti-FLAG M2 antibody (Sigma-Aldrich F1804, Lot SLCJ3741; 1:1000); co-culture specificity experiment used anti-mouse CD19-Alexa Fluor 700 antibody (ThermoFisher 56-0193-82, Lot 2869684; 1:100).

Validation	Antibodies were purchased from commercial sources and have been validated by the manufacturers. Verification statements and literature citations can be found at the manufacturers' websites:<br>Anti-FLAG M2 antibody: https://www.sigmaaldrich.com/US/en/product/sigma/f1804<br>Anti-mouse CD19-Alexa Fluor 700 antibody: https://www.thermofisher.com/antibody/product/CD19-Antibody-clone-eBio1D3-1D3-Monoclonal/56-0193-82

Eukaryotic cell lines

Policy information about cell lines and Sex and Gender in Research

Cell line source(s)	Cell lines from ATCC: A20, A431, A549, Jurkat<br>Cell lines from ThermoFisher: HEK293FT<br>Cell lines from colleagues: BJ-HA (BJ fibroblasts stably displaying HA tag)
Authentication	None of these cell lines were authenticated prior to use.
Mycoplasma contamination	None of these cell lines were tested for Mycoplasma prior to use.
Commonly misidentified lines<br>(See ICLAC register)	No commonly misidentified cell lines were used in this study.

Animals and other research organisms

Policy information about studies involving animals; ARRIVE guidelines recommended for reporting animal research, and Sex and Gender in Research

Laboratory animals	Specific pathogen-free facilities at the Broad Institute was used for the storage and care of all mice. Mice were housed at a temperature of 67–73 °F, relative humidity of 30–60%, and maintained in a 12 h light–dark cycle. Female C57BL/6J mice (aged 8–12 weeks) were obtained from the Jackson Laboratory (strain 000664). All mice were maintained on a 12-h light:dark cycle with ad libitum access to food and water.
Wild animals	No wild animals were used in this study.
Reporting on sex	As we did not expect a difference in PVC activity between males and females (since all endogenous receptors targeted in this study are present in both males and females), PVC injections were restricted to female animals.
Field-collected samples	We did not collect samples from the field.
Ethics oversight	All mouse experiments conformed to guidelines established by the National Institutes of Health and were conducted under protocols approved by the Institutional Animal Care and Use Committees (IACUC) of the Broad Institute of MIT and Harvard.

Note that full information on the approval of the study protocol must also be provided in the manuscript.

Plants

Seed stocks	N/A - no plants were used in this study.
Novel plant genotypes	N/A - no plants were used in this study.
Authentication	N/A - no plants were used in this study.

Flow Cytometry

Plots

Confirm that:

- [x] The axis labels state the marker and fluorochrome used (e.g. CD4-FITC).
- [x] The axis scales are clearly visible. Include numbers along axes only for bottom left plot of group (a 'group' is an analysis of identical markers).
- [x] All plots are contour plots with outliers or pseudocolor plots.
- [x] A numerical value for number of cells or percentage (with statistics) is provided.

Methodology

Sample preparation	Cells were harvested by incubation with 30 µL of TrypLE Express dissociation reagent (ThermoFisher 12604) for 5 min at RT followed by resuspension with 50 µL of cold flow cytometry buffer [PBS supplemented with 2% EDTA (Life Technologies 15575020) and 5% FBS (VWR 97068-085)]. Additional information can be found in the Methods under "Flow cytometry" and "Specificity assay with mixed populations".
Instrument	Samples were run on a Beckman Coulter Cytoflex S flow cytometer.
Software	Analysis was performed using FlowJo (10.8.2).
Cell population abundance	Post-sort fractions were not analyzed.
Gating strategy	The gating scheme with original axis labels can be found in Extended Data Fig. 3g. We defined A20 and A431 populations using flow cytometry data from untreated co-cultures; the threshold was set roughly halfway between the two clusters of cells (low FSC/high A700 vs. high FSC/low A700) and then applied to experimental data.

☒ Tick this box to confirm that a figure exemplifying the gating strategy is provided in the Supplementary Information.
