# Supplementary material for: Targeted delivery of diverse biomolecules with engineered bacterial nanosyringes
Source: Nat Biotechnol. 2025 Aug 12;44(7):1121–5. doi: 10.1038/s41587-025-02774-x (PMC13368577; doi:10.1038/s41587-025-02774-x)

Extended Data Fig. 1d

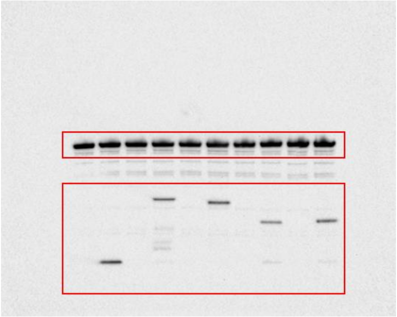

Extended Data Fig. 2a (Raw)

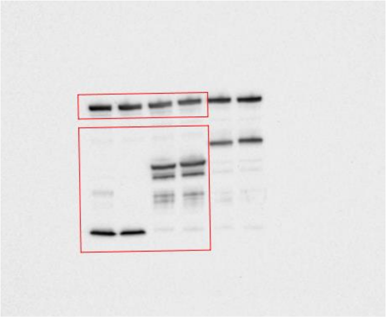

Extended Data Fig. 2a (Purified)

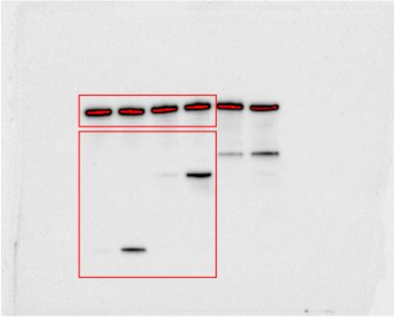

Extended Data Fig. 2b
(ssODN)

Extended Data Fig. 2b
(ssODN +HUHe site)

(Cy3)

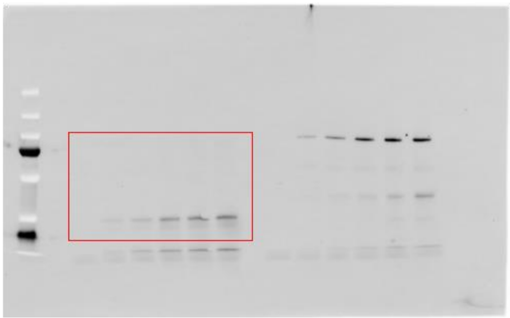

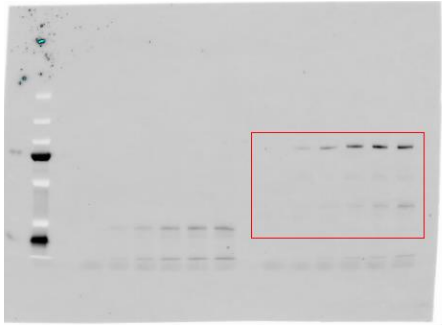

(α-FLAG)

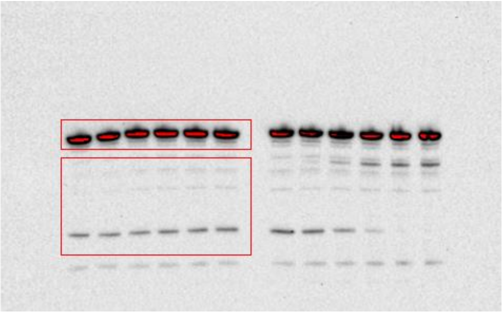

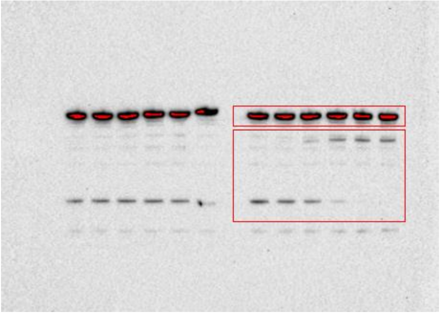

Extended Data Fig. 3b

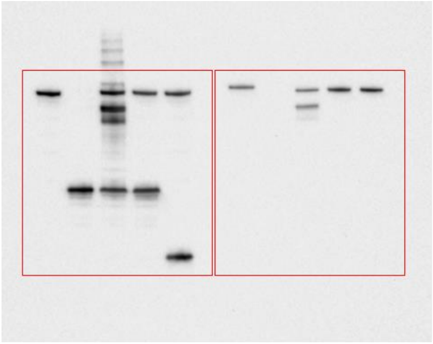

Supplement: Supplementary file 6 — Unprocessed blot images. [file 41587_2025_2774_MOESM6_ESM.pdf]
