# Supplementary material for: Targeted delivery of diverse biomolecules with engineered bacterial nanosyringes
Source: Nat Biotechnol. 2025 Aug 12;44(7):1121–5. doi: 10.1038/s41587-025-02774-x (PMC13368577; doi:10.1038/s41587-025-02774-x)
Supplement: Supplementary file 2 — Peer Review File [file 41587_2025_2774_MOESM2_ESM.pdf]

nature portfolio

Peer Review File

Targeted delivery of diverse biomolecules with engineered bacterial nanosyringes

Corresponding Author: Professor Feng Zhang

Parts of this Peer Review File have been redacted as indicated as we could not obtain permission to publish the reports from one of the peer reviewers.

This file contains all reviewer reports in order by version, followed by all author rebuttals in order by version.

Version 0:

Reviewer comments:

Reviewer #1

(Remarks to the Author)
This manuscript focuses on engineering the Photorabdus virulence cassette (PVC) to deliver Cas9 RNP and ssDNA into cells. A methodology is developed to conjugate targeting proteins to the tip of the PVC. Engineered PVCs were able to deliver Cas9 RNP and ssDNAs into mammalian cells in vitro, although with low efficiency in comparison to other well established methods, such as lipid nanoparticles (LNPs). PVCs could also be targeted to cells selectively by conjugating targeting ligands to the tip of the PVC.
Although this paper does expand the capabilities of PVCs it lacks the significance needed for publication in Nature Biotechnology. In particular, it is unclear if the PVC technology has any significant advantage over existing delivery methods, such as LNPs, which already work well in multiple organs in vivo. In addition, PVCs will be antigenic and cannot be administered multiple times. A key piece of data missing from this paper is in vivo demonstration of delivery efficacy.

Reviewer #2

(Remarks to the Author)
Summary:

In this work, Kreitz J. et al. modify the extracellular contractile injection system known as PVCs into a more modular protein delivery tool which they term as SPEAR. The authors have previously published a paper reprogramming the PVCs into a protein delivery system (https://www.nature.com/articles/s41586-023-05870-7) and in this manuscript, they have expanded the type of cargoes that can be delivered by fusing proteins to the spike tip of the system in a similar manner to what has been previously shown in T6SSs. Additionally, the authors show that the specificity of the PVCs can be changed in vitro via the SpyCatcher/SpyTag system, where the purified particles can be mixed with target binding proteins including DARPins, nanobodies, and antibodies which can allow for specific cell targeting in a mixed cell context.

The results of this paper are exciting, and they address the concern that only proteinaceous cargo may be delivered by engineered eCIS. It also furthers the modularity of the systems for higher orders of customization via modification of purified particles in vitro. Below are our comments that should be addressed before publication.

Major comments:

*Fig. 1c: Is it possible to (semi-)quantify the negative stain EM data shown in Fig. 1c? How often do the authors observe the matching number of spike-associated densities for a given sample. This insight would be important also to interpret the data shown in Fig. 1d.

*Fig. 1d: The RT-qPCR results indicate a similar level of RNA for the Pvc10-Cas9RNP, Cas9RNP-Pvc10-Cas9RNP, and the Pvc8-Cas9RNP. Since there is one Cas9RNP Pvc10 protein per particle, two in the Cas9RNP-Pvc10-Cas9RNP, and three Pvc8-Cas9RNP per particle, why do we not see a corresponding difference in the level of RNA abundance between the different samples? Is a ~5-log enrichment (line 42) consistent with what one would expect from having 1-3 binders on the

spike?

*Fig. 1e-g: Untargeted PVCs are used as a control for the experiments. The experiments convincingly show the desired effect of the engineered and targeted PVCs in comparison to the untargeted version. That said, even after their Kreitz et al. 2023 paper, some researchers in the field speculate whether or not the binding of targeted PVCs could trigger their uptake and lead to the desired effect even in the absence of contraction. It has so far not been shown whether or not injection is actually required for cargo delivery. The way to answer this question would be an experiment in which a non-contractile (but targeted) PVC version would be used as control. Such non-contractile CISs have been engineered previously (e.g. PMIDs 29222345 or 36894633) and could be designed accordingly for PVCs. Even though the outcome of this experiment would not jeopardize or question the main conclusions of the paper (that PVCs are a valuable biotechnological tool), the experiment would clarify the mechanism of delivery, which is an important factor for future research and application of PVCs.

Minor comments:

*Line 28: Even though it is shown in Extended Figure 1a, it should be mentioned in the text that the native tube-delivered payloads have been deleted in the used constructs.

*In general, there needs to be more detail in either in the text or in the figure legends for the experiments/results being presented in the main figures. In Fig. 2b for example, it is neither stated in the text nor the figure legend what is being injected nor why the cells become GFP+.

*In order to differentiate between Pvc10 and Pvc8 in the text, we suggest referring to Pvc10 as the “tip” and Pvc8 as the “spike” throughout the text in lieu of 2 spike proteins. This matches the equivalent proteins in T6SSs better and will help to limit confusion amongst readers.

*In line 340, it is stated that long term storage of PVCs is done with 5% glycerol at -80 oC. Were these PVCs tested and do they have a similar level of activity to non-frozen samples?

*Line 16: add extracellular to CIS, eCIS

*Lines 197-205: In Fig. 1 figure legends, the PVC(T)/PVC(UT) explanation needs to be moved from g to e, where it is first introduced.

*Line 209: Remove spontaneous

*Line 355: The O in complete should not be capitalized

Reviewer #3

(Remarks to the Author)
The manuscript “Delivery of multimodal cargos using an engineered bacterial injection system” is a very creative and interesting report submitted in the Brief Communication format. The studies are well done and clear. The current study could be improved by demonstrating (1) in vitro insertion of a therapeutically relevant gene, and demonstrating (2) in vivo proof-of-concept activity. I believe this study may be publishable in Nature Biotechnology as a brief communication after revisions.

1. The step described on page 3 and demonstrated in Figure 2f is a key advance in my opinion. Here, PVCs loaded both with Cas9 RNPs and HUHe-ssODNs produced both Cas9-mediated indels and ssODN-mediated DNA insertions in untransfected HEK293FT cells. The authors further show that this approach can work in other cell types such as BJ and Jurkat. Given the technology and translational audience of Nat. Biotech., it would be worthwhile to combine these pursuits and demonstrate DNA insertion of a therapeutically relevant gene in cells. For example, could a CAR be inserted into human T cells ex vivo? I suggest addition of this data to help the study reach the impact and audience of the journal.

2. The enclosed manuscript builds and improves upon Ref. 13 (Kreitz et al. Nature 616, 357–364 (2023)) in many ways. In the Nature article, it was shown that first generation PVCs could deliver Cre protein into the brains of Ai9 (loxP-stop-loxP tdTomato) mice to produce tdTomato signal in the hippocampus. An in vivo demonstration of this current improved PVC designs appears to be missing. I suggest to use the Ai9 mice again and show that SPEAR works in vivo. Could be brain, muscle, spleen, liver, or any cells/tissue targetable by the new design.

3. In the landscape of viral and nonviral delivery systems, how can readers frame and understand SPEAR? There are many highly efficient ways to transfect cells, and it was unclear if, or how, SPEAR can be leveraged to do things that current approaches cannot already do. If the advance centers only on creativity and engineering, and not translational science, this can be stated as well.

4. It is currently unclear, particularly with the brief communication format, what are the broader impacts and potential utility of SPEAR. I suggest adding text to detail broader impacts and comments on how SPEAR could be used therapeutically or otherwise find utility.

Author Rebuttal letter:

Response to reviewer comments for manuscript NBT-BC64902
Multimodal cargo delivery with an engineered bacterial injection system
Kreitz et al.

We are grateful to the reviewers for their evaluation of our manuscript and for their helpful feedback. The revised manuscript contains additional data and discussion to address the reviewers' comments; these changes are summarized in the point-by-point response below.

Reviewer #1:

This manuscript focuses on engineering the Photorabdus virulence cassette (PVC) to deliver Cas9 RNP and ssDNA into cells. A methodology is developed to conjugate targeting proteins to the tip of the PVC. Engineered PVCs were able to deliver Cas9 RNP and ssDNAs into mammalian cells in vitro, although with low efficiency in comparison to other well established methods, such as lipid nanoparticles (LNPs). PVCs could also be targeted to cells selectively by conjugating targeting ligands to the tip of the PVC. Although this paper does expand the capabilities of PVCs it lacks the significance needed for publication in Nature Biotechnology. In particular, it is unclear if the PVC technology has any significant advantage over existing delivery methods, such as LNPs, which already work well in multiple organs in vivo.

We thank the reviewer for their time in reviewing our manuscript. In our second submission we have provided substantial additional validation of spike-based cargo delivery (Extended Data Fig. 1e-i), an additional figure dedicated to in vivo experiments (Extended Data Fig. 4), and improvements to the text and figures to increase clarity and better convey the broader implications of SPEAR in the context of other delivery technologies.

In general, we do not argue that SPEAR is competitive with LNPs in terms of raw delivery efficiency in its current form; rather, we believe this new technology offers specific advantages which make it attractive for a subset of potential delivery tasks. Specifically, we believe the system offers the following advantages:

Modularity: Due to the unique structure of the PVC, targeting and cargo loading take place in structurally independent locations within the complex, such that both can be tuned independently of the other. In addition, we have now demonstrated two separate cargo loading mechanisms for this system (tube-based loading and spike-based loading), which can be used in tandem, allowing mixtures of defined cargos to be delivered simultaneously (Fig. 1g).

Cargo flexibility: We have now demonstrated that PVCs can be loaded with a wide variety of molecular cargos (proteins, RNPs, ssDNA, and even combinations of all three). This system does not appear limited by cargo size constraints (as with many other vectors, such as AAVs) and is not limited to only one category of cargo (protein, RNA, or DNA) as with many other vectors. We are exploring the possibility of leveraging SPEAR to help internalize much larger cargos, including VLPs1, protein nanoparticles2, or DNA origami cages3, many of which lack native mechanisms for membrane transit or endosomal escape.

Delivery of folded cargos: Most existing protein delivery systems unfold cargos prior to delivery. However, because many proteins cannot spontaneously re-fold once inside the target cell, technologies capable of delivering folded proteins are highly sought-after. Notably, we have shown that SPEAR can translocate cargos directly through the cellular membrane without loss of native folding, allowing for single-step delivery of macromolecular assemblies (Cas9 RNPs).

Target specificity: We have shown that PVC activity is tightly regulated and requires specific recognition of a target receptor by the tail fiber (Pvc13). Notably, PVC activity is thought to require binding by multiple tail fibers simultaneously—in contrast to many other vectors, in which a single binding event is often sufficient to trigger internalization—resulting in an impressive level of target specificity for this system (see Fig. 3 from our 2023 paper for a more detailed analysis of PVC specificity).

Efficient production and functionalization: Because PVCs can be efficiently purified from bacteria, and can then be retargeted and loaded with cargos in vitro (Fig. 2 of this manuscript), we believe this system could be efficiently scaled up for downstream manufacturing.

Novel mechanism of action: Finally, we argue SPEAR makes a meaningful contribution to the delivery space because it offers a fundamentally novel mechanism of action that has not been exploited by other vector technologies. This mechanism—direct membrane

puncture—does not rely on native pathways for internalization, cellular trafficking, or host transcription/translation (as with many other vectors, which largely rely on receptor-mediated endocytosis or membrane fusion for entry), thus making it an ideal candidate for use in cell types and tissues which are metabolically inactive or senescent (a longstanding challenge in delivery) or in completely different organisms which are otherwise difficult to access with current delivery tools (e.g. plants, single-celled parasites, or even prokaryotes). We believe the contribution of a new category of vectors based on membrane-puncturing contractile injection systems will offer ample opportunity for future work to optimize delivery efficiency and further expand the potential application space for this new platform.
In summary, we believe SPEAR offers a favorable combination of cargo flexibility, target specificity, and modularity/programmability which sets it apart from existing delivery technologies. Whereas other delivery vehicles, such as AAVs and LNPs, have benefitted from years of development and optimization, PVCs represent a fundamentally novel technology with a radically different mechanism of action. In this study, we established several powerful new methods to functionalize PVC cargo loading and cellular targeting; we are optimistic that future studies will be able to leverage these approaches to demonstrate new therapeutic applications for these tools and increase their efficiency in cells.
We have added the following text to the revised manuscript to guide the reader's interpretation of the broader impacts of SPEAR in the context of therapeutic delivery:

- Lines 119-124: Added a discussion of the benefits of SPEAR and potential downstream applications for this novel technology.
- Lines 106-107: After our summary of the novel in vivo data, we added a statement of the potential utility of SPEAR in gene therapies targeting B cells.
- Lines 89-91: Added an expanded explanation of the utility of ST/SC-based in vitro retargeting.

In addition, PVCs will be antigenic and cannot be administered multiple times.

To directly test for the possibility of immune clearance in vivo, we performed a neutralization assay using mouse serum following PVC challenge (taking inspiration from other studies which have used a similar experiment to understand immune clearance4,5; see the new section entitled "Serum neutralization assay" in the Methods). Briefly, we injected mice (i.v.) with either a mock injection (PBS) or MHC-II targeting PVCs (retargeted in vitro as in Fig. 2j); PVCs were introduced either once (at t=0) or twice (at t=0 and t=7 days) to allow the animals to mount an effective neutralizing antibody response. Serum was then extracted at t=14 days, incubated with an identical PVC sample, and titrated against A20 cells to understand the effect of pre-challenged serum on PVC activity. The results can be found in the new Extended Data Fig. 4d; although there was a detectable drop in delivery efficiency—potentially indicating the presence of anti-PVC neutralizing antibodies in the serum—the observed reduction in activity was relatively minor (IC50 dropped from around 3.5 ng/μL to 4 ng/μL and 4.5 ng/μL for the 1-dose serum and 2-dose serum, respectively, representing a ~15% and ~30% reduction in potency). This indicates that although PVCs likely trigger an adaptive immune response in vivo—as correctly suggested by the reviewer—this response is insufficient to abolish PVC function. This is likely because the region most likely to be antigenic—the PVC sheath—is probably highly tolerant to binding by neutralizing antibody, since we have previously shown that the sheath can be decorated with novel protein domains without loss of PVC injection activity. Overall, this result demonstrates that PVCs likely are antigenic, but to a level that is unlikely to completely preclude subsequent injections.

A key piece of data missing from this paper is in vivo demonstration of delivery efficacy.

To investigate the activity of SPEAR in vivo, we sought to recapitulate the result from Fig. 2j (in vitro reprogramming of PVC target specificity) to selectively suppress a defined cell population within live mice (rather than within artificial mixed populations in cell culture). Briefly, we reprogrammed toxin-loaded PVCs to target mouse MHC-II using the ST/SC-based in vitro retargeting method described in Figs. 2g-j, introduced the resulting retargeted particles into mice via systemic i.v. injections (representing a departure from all previous in vivo studies of PVCs, including our 2023 paper on this topic, which used local injections), extracted splenocytes after 24hrs, and analyzed the resulting cells with flow cytometry (see the new "In vivo B cell depletion assay" section in the Methods). The results can be found in the new Extended Data Fig. 4a-c; we found that in vitro-retargeted PVCs produced specific cytotoxicity against MHC-II+ cell types—including B cells—within 24hr after i.v. injection in mice. In total, PVC treatment achieved roughly 7% bulk activity in MHC-II+ cells in circulation after a single dose, a level that very likely exceeds the therapeutic threshold for many applications (including B cell-based therapeutic secretion6). This result demonstrates (1) that PVCs are functional in circulation (building on our 2023 manuscript, which only showed in vivo activity via local stereotaxic injection in the brain); and (2) that in vitro-reprogrammed PVCs retain their desired specificity after introduction into animals (confirming the in vitro result in Fig. 2j). This result also raises the possibility of using PVCs for therapeutic

interventions targeting B cells.

We thank the reviewer for their time in reading our manuscript and for their comments.
Reviewer #2:

Summary:

In this work, Kreitz J. et al. modify the extracellular contractile injection system known as PVCs into a more modular protein delivery tool which they term as SPEAR. The authors have previously published a paper reprogramming the PVCs into a protein delivery system (https://www.nature.com/articles/s410-7) and in this manuscript, they have expanded the type of cargoes that can be delivered by fusing proteins to the spike tip of the system in a similar manner to what has been previously shown in T6SSs. Additionally, the authors show that the specificity of the PVCs can be changed in vitro via the SpyCatcher/SpyTag system, where the purified particles can be mixed with target binding proteins including DARPins, nanobodies, and antibodies which can allow for specific cell targeting in a mixed cell context.

The results of this paper are exciting, and they address the concern that only proteinaceous cargo may be delivered by engineered eCIS. It also furthers the modularity of the systems for higher orders of customization via modification of purified particles in vitro. Below are our comments that should be addressed before publication.

We thank the reviewer for their feedback. In our revised manuscript we have provided substantial additional validation of spike-based cargo delivery (Extended Data Fig. 1e-i), an additional figure dedicated to in vivo experiments (Extended Data Fig. 4), and improvements to the text and figures to increase clarity and better convey the details of the experiments performed in this study.

Major comments:

*Fig. 1c: Is it possible to (semi-)quantify the negative stain EM data shown in Fig. 1c? How often do the authors observe the matching number of spike-associated densities for a given sample. This insight would be important also to interpret the data shown in Fig. 1d.

We have provided additional EM images at lower magnification to provide a broader view of these samples; these images can be found in the new Extended Data Fig. 1e. In addition, we performed a semi-quantitative analysis of Cas9 loading efficiency (also in Extended Data Fig. 1e). We observed that the novel designs did not load cargos at equivalent efficiencies: Pvc8-Cas9 was most efficiently loaded (83%), followed by Pvc10-Cas9 (55%), Cas9-Pvc10-Cas9 (43%), and Cas9-Pvc10 (38%). Interestingly, the variations in loading efficiency between the Pvc10 designs roughly correlated with observed variations in delivery efficiency (Fig. 1e)—Pvc10-Cas9 produced the highest efficiency (~6%), followed by Cas9-Pvc10-Cas9 (~4.5%) and Cas9-Pvc10 (~3%)—suggesting delivery efficiency is affected by how efficiently cargos are loaded onto the PVC complex. The high loading efficiency for Pvc8-Cas9 is likely a product of the fact that Pvc8 is a core structural protein for PVCs (unlike Pvc10) and is required for proper PVC assembly (Jiang et al. 2019 Cell7); Pvc10, by contrast, may simply dissociate from the PVC complex over time, resulting in lower loading efficiencies. In general, we believe the reported loading efficiencies in Extended Data Fig. 1e are underestimates, as our counting scheme was highly conservative and counted ambiguous complexes as unloaded (Pvc8-Cas9, for instance, probably loaded Cas9 in 100% of particles, given the structural importance of Pvc8 in PVC assembly). Nonetheless, we think this analysis provides valuable context for the activity observed in Fig. 1, and guides best practices for downstream application of this technology (e.g. fusing Pvc10 to the N-terminus of the cargo is clearly preferable to a C-terminal fusion).

*Fig. 1d: The RT-qPCR results indicate a similar level of RNA for the Pvc10-Cas9RNP, Cas9RNP-Pvc10-Cas9RNP, and the Pvc8-Cas9RNP. Since there is one Cas9RNP Pvc10 protein per particle, two in the Cas9RNP-Pvc10-Cas9RNP, and three Pvc8-Cas9RNP per particle, why do we not see a corresponding difference in the level of RNA abundance between the different samples? Is a ~5-log enrichment (line 42) consistent with what one would expect from having 1-3 binders on the spike?

To facilitate comparison between the gRNA levels of PVC designs harboring Cas9 RNPs, we have re-plotted the data from Fig. 1d on a linear scale; this can be found in Extended Data Fig. 1f. Contrary to the log-scale plot from Fig. 1d (which implied the gRNA levels were equivalent), this new linear-scale plot makes clear that there were differences in gRNA loading efficiency which roughly corresponded both to (1) RNP loading efficiency (as reported in Extended Data Fig. 1e); and (2) RNP multiplicity (i.e. how many RNPs are theoretically loaded per complex). We note the following observations:

Pvc10-Cas9 loaded more gRNA than Cas9-Pvc10, confirming the result observed in ED Fig. 1e (in which Pvc10-Cas9 loaded more efficiently than Cas9-Pvc10).

Although Cas9-Pvc10-Cas9 would be expected to load twice as much gRNA as either Pvc10-Cas9 or Cas9-Pvc10 (given each loaded complex possesses two RNPs), we now know from ED Fig. 1e that this dual-Cas9 design loaded significantly less efficiently than Pvc10-Cas9 and slightly more efficiently than Cas9-Pvc10. This insight provides context for these three designs' relative gRNA levels: we observed that Cas9-Pvc10-Cas9 loaded significantly more gRNA than Cas9-Pvc10, but only slightly more gRNA than Pvc10-Cas9.

As expected both by its high cargo loading efficiency (ED Fig. 1e) and by the fact that each loaded PVC particle theoretically houses 3 RNPs (given Pvc8 assembles as a trimer), Pvc8-Cas9 loaded the most gRNA of all designs tested. However, this design only empirically loaded slightly more gRNA than Cas9-Pvc10-Cas9; this could suggest that many Pvc8-Cas9 PVCs visualized in ED Fig. 1e and Fig. 1c actually carry Cas9 apoprotein (lacking gRNA), perhaps as a result of the increased steric strain of three separate Cas9 monomers in close proximity to the spike complex (see the predicted structure in ED Fig. 1b and EM images in Fig. 1c and ED Fig. 1e). This could reconcile the observation that Pvc8-Cas9, despite ostensibly loading the most cargos, actually yields the worst delivery activity (Fig. 1e) of all designs tested.

Although we feel it is still important to depict the result from Fig. 1d on a log scale (to accurately portray the magnitude of difference in gRNA levels between WT spike and Cas9-loaded spikes) a linear scale depiction (ED Fig. 1f) helps to compare the relative gRNA levels of Cas9-loaded PVC designs and provides valuable context for the differences in cargo loading efficiency and delivery activity observed between them. Thus, we opted to include both plots in the revised manuscript.

*Fig. 1e-g: Untargeted PVCs are used as a control for the experiments. The experiments convincingly show the desired effect of the engineered and targeted PVCs in comparison to the untargeted version. That said, even after their Kreitz et al. 2023 paper, some researchers in the field speculate whether or not the binding of targeted PVCs could trigger their uptake and lead to the desired effect even in the absence of contraction. It has so far not been shown whether or not injection is actually required for cargo delivery. The way to answer this question would be an experiment in which a non-contractile (but targeted) PVC version would be used as control. Such non-contractile CISs have been engineered previously (e.g. PMIDs 29222345 or 36894633) and could be designed accordingly for PVCs. Even though the outcome of this experiment would not jeopardize or question the main conclusions of the paper (that PVCs are a valuable biotechnological tool), the experiment would clarify the mechanism of delivery, which is an important factor for future research and application of PVCs.

To investigate whether the observed PVC activity is dependent on sheath contraction, we generated two new non-contractile PVC mutants based on the design used in Casu et al. (2023) (each mutant containing a 5aa insertion within the "handshake" region of the sheath; see the new sequence entries in Supplementary Tables 4-5) and re-ran the experiment shown in Fig. 1f; the result can be found in Extended Data Fig. 1h. Excitingly, we found that these mutant PVCs exhibited significantly depleted activity (~60-80% reduction with non-contractile versions of Pvc10-Cas9 and Pvc8-Cas9), suggesting the activity observed in unmutated particles likely involved sheath contraction. Interestingly, these mutants failed to completely abolish activity; one possible explanation is that they didn't fully inhibit sheath contraction, as we observed under TEM what we believe to be several partially contracted sheaths (see EM image below; contracted portion of the sheath marked with an arrow):

The presence of partially-contracted PVCs could imply that the designs we used didn't completely inhibit sheath contraction, thus potentially accounting for the residual activity produced by these mutants. We are interested in exploring additional mutants which may more completely inhibit sheath contraction; for instance, insertions of more than 5aa in the handshake region of the sheath. Nonetheless, we believe the observed 60-80% reduction in activity in our current mutants provides a strong indication that sheath contraction is involved in the activity observed in this study.

Separately, another way we've attempted to show that the observed activity was the result of injection by the PVC (rather than through other mechanisms, such as passive uptake) was through the use of ΔPvc10 controls (used in this manuscript as well as our 2023 paper). In principle, if the observed activity were the result of cellular binding alone, then properly targeted PVCs lacking Pvc10 should produce comparable activity to unmutated particles, since Pvc10 is not necessary for the assembly of the PVC (as shown by Jiang et al. 2019 Cell7) and is unlikely to mediate cellular binding by itself (its cone-like structure instead suggests it "sharpens" Pvc8). However, if the activity were instead the result of injection of the spike through the membrane by the PVC complex, then ΔPvc10 particles may show depleted signal, since these particles likely possess "blunted" spikes which may be unable to efficiently puncture the cellular membrane. In our 2023 paper, we showed that ΔPvc10 mutations completely abolished the activity of internal (tube-loaded) PVCs both in vitro and in vivo. In

addition, in this manuscript, we showed that PVCs harboring Pvc8-Cas9 require Pvc10 to produce detectable indels in human cells (Extended Data Fig. 1g) and that ΔPvc10 PVCs loaded via the tube are inactive until complemented with separately-purified Pvc10 protein (Fig. 2b). These results indicate that PVC activity—both with internal cargos and spike-cargo fusions—requires a Pvc10-tipped spike complex, and is therefore likely to involve membrane penetration in some way.

In summary, we believe the two experiments presented here (with non-contractile PVCs and ΔPvc10 PVCs) provide sufficient evidence that the observed activity is dependent on an injection-based mechanism.

Minor comments:

*Line 28: Even though it is shown in Extended Figure 1a, it should be mentioned in the text that the native tube-delivered payloads have been deleted in the used constructs.

We have added a mention of deleted native cargos in line 30.

*In general, there needs to be more detail in either in the text or in the figure legends for the experiments/results being presented in the main figures. In Fig. 2b for example, it is neither stated in the text nor the figure legend what is being injected nor why the cells become GFP+.
In the revised manuscript, we have attempted to improve the clarity of our experiments by providing additional descriptive details, particularly in the figure legends.
 Details about the experiment in Fig. 2b were added to both the figure legends and the text.
 Further detail about the ZFD experiment in Fig. 1g was added to the main text and figure legends.
 Multiple mentions of “human cells” were replaced by the actual cell lines used (generally HEK293FT).
 We expanded and/or re-worded the figure legends for Fig. 2d-f to improve the clarity of the ssDNA delivery experiment.
 A significantly expanded Fig. 2j figure legend to describe the population editing experiment was provided.
 A re-worded description of the experiment in Extended Data Fig. 5 (lines 111-114) to more clearly describe the design of the PVC used in this experiment as well as the result.

*In order to differentiate between Pvc10 and Pvc8 in the text, we suggest referring to Pvc10 as the “tip” and Pvc8 as the “spike” throughout the text in lieu of 2 spike proteins. This matches the equivalent proteins in T6SSs better and will help to limit confusion amongst readers.

We have standardized the terms used to refer to Pvc10 and Pvc8 throughout the manuscript. All uses of “spike” now exclusively refer to Pvc8, and all uses of “spike tip” refer to Pvc10. References to the complex of Pvc8 and Pvc10 now use “spike complex”.

*In line 340, it is stated that long term storage of PVCs is done with 5% glycerol at -80 oC. Were these PVCs tested and do they have a similar level of activity to non-frozen samples?

We have routinely stored PVC samples at -80C in 5% glycerol/PBS without significant loss of efficiency. To confirm this, for this revision we measured the activity of two previously frozen PVC protein samples alongside freshly-purified samples of the same designs:
1. Tube-loaded PVCs (frozen in April 2023)
2. Spike-loaded PVCs (frozen in March 2024)
The results can be found in the new Extended Data Fig. 1i; In short, we found that frozen tube-loaded PVCs exhibited roughly equivalent activity to freshly purified samples after 2 years at -80C, and that the frozen spike-loaded PVCs exhibited a relatively minor (~25%) drop in activity after a year at -80C. These results demonstrate that PVCs of both designs can be effectively stored for long durations via freezing at -80C. We also note that a small amount of glycerol (5% v/v for this experiment) was essential; PVCs frozen in PBS alone exhibited drastically depleted activity. We finally note that PVC samples were added to the freezer directly (flash-freezing was not used); we have added mention of this in the Methods.

*Line 16: add extracellular to CIS, eCIS
*Lines 197-205: In Fig. 1 figure legends, the PVC(T)/PVC(UT) explanation needs to be moved from g to e, where it is first introduced.

*Line 209: Remove spontaneous

*Line 355: The O in complete should not be capitalized

This protease inhibitor cocktail is actually called “cOmplete” by the manufacturer; we have

updated the text to provide the manufacturer's product number to help readers find this reagent.

All other minor comments have been addressed in our revised manuscript.

We thank the reviewer for their time in evaluating our manuscript and for their helpful comments!
Reviewer #3:

The manuscript "Delivery of multimodal cargos using an engineered bacterial injection system" is a very creative and interesting report submitted in the Brief Communication format. The studies are well done and clear. The current study could be improved by demonstrating (1) in vitro insertion of a therapeutically relevant gene, and demonstrating (2) in vivo proof-of-concept activity. I believe this study may be publishable in Nature Biotechnology as a brief communication after revisions.

We thank the reviewer for their time in reviewing our manuscript. In our second submission we have provided substantial additional validation of spike-based cargo delivery (Extended Data Fig. 1e-i), an additional figure dedicated to in vivo experiments (Extended Data Fig. 4), and improvements to the text and figures to increase clarity and better convey the broader implications of SPEAR in the context of other delivery technologies.

1.The step described on page 3 and demonstrated in Figure 2f is a key advance in my opinion. Here, PVCs loaded both with Cas9 RNPs and HUHe-ssODNs produced both Cas9-mediated indels and ssODN-mediated DNA insertions in untransfected HEK293FT cells. The authors further show that this approach can work in other cell types such as BJ and Jurkat. Given the technology and translational audience of Nat. Biotech., it would be worthwhile to combine these pursuits and demonstrate DNA insertion of a therapeutically relevant gene in cells. For example, could a CAR be inserted into human T cells ex vivo? I suggest addition of this data to help the study reach the impact and audience of the journal.

While we agree that delivery of ORF-length DNA cargos—such as CARs—using SPEAR would represent a highly exciting translational application for this technology, we have several important concerns about the feasibility of such an experiment:
1. PVCs likely cannot deliver ORF-length ssDNA due to intrinsic biophysical properties of DNA. Since a strand of DNA is roughly 3.4 Å/nt, a ssDNA CAR cargo which is 3,000 nt in length (including the ORF, regulatory elements, and flanking homology arms) would be approximately 1 μm in length—roughly 10x the average length of a PVC particle (~116 nm)7,8. Given the hypothesized mechanism of PVC-mediated delivery (depicted in Extended Data Fig. 1c), in which spike-loaded cargos are "pulled" into cells via mechanical injection of the spike through the membrane, we think it is unlikely that PVCs will be able to successfully inject cargos exceeding the size of the PVC itself (especially by such a wide margin).
2. HDR-mediated insertion of ORF-length ssDNA is less efficient than insertion of oligo-length ssDNA, likely pushing insertion efficiencies below the detection limit. It has been reported elsewhere that the efficiency of HDR-mediated gene insertion is inversely correlated to the length of the insert9. Given the level of background insertions in Fig. 2f (likely due to incomplete removal of unloaded ssDNA from PVC conjugation reactions), transitioning from oligo-length ssDNA cargos to ORF-length ssDNA cargos would likely push the insertion efficiency below background.
Given these concerns, we believe it would be challenging to successfully deliver ORF-length DNA cargos with SPEAR in its current form. However, we note that the proof of concept with oligo-length ssDNA presented in Fig. 2d-f still presents powerful opportunities for therapeutic application, particularly in gene therapy. For instance, since the insertions in Fig. 2f were 6 nt in length (with 40nt of homology on each flank of the insert), this technology is already technically capable of addressing the ~80% of rare diseases which result from single amino acid mutations10. We envision that—in the short run—SPEAR-based ssDNA delivery will be better suited for applications in which small DNA insertions are required (for instance, in gene correction) rather than in delivery of long, ORF-length DNAs.

2.The enclosed manuscript builds and improves upon Ref. 13 (Kreitz et al. Nature 616, 357–364 (2023)) in many ways. In the Nature article, it was shown that first generation PVCs could deliver Cre protein into the brains of Ai9 (loxP-stop-loxP tdTomato) mice to produce tdTomato signal in the hippocampus. An in vivo demonstration of this current improved PVC designs appears to be missing. I suggest to use the Ai9 mice again and show that SPEAR works in vivo. Could be brain, muscle, spleen, liver, or any cells/tissue targetable by the new design.

To investigate the activity of SPEAR in vivo, we sought to recapitulate the result from Fig. 2j (in vitro reprogramming of PVC target specificity) to selectively suppress a defined cell population within live mice (rather than within artificial mixed populations in cell culture).

Although we considered the possibility of repeating the brain injections used in our 2023 manuscript (as suggested by the reviewer), we felt it would be more powerful to demonstrate PVC activity in circulation (i.e. via i.v. injection), where immunity and dilution effects would be more of a challenge. Briefly, we reprogrammed toxin-loaded PVCs to target mouse MHC-II using the ST/SC-based in vitro retargeting method described in Figs. 2g-j, introduced the resulting retargeted particles into mice via systemic i.v. injections (representing a departure from all previous in vivo studies of PVCs, which used local injections), extracted splenocytes after 24hrs, and analyzed the resulting cells with flow cytometry (see the new “In vivo B cell depletion assay” section in the Methods). The results can be found in the new Extended Data Fig. 4a-c; we found that in vitro-retargeted PVCs produced specific cytotoxicity against MHC-II+ cell types—including B cells—within 24hr after i.v. injection in mice. In total, PVC treatment achieved roughly 7% bulk activity in MHC-II+ cells in circulation, a level that very likely exceeds the therapeutic threshold for many applications (including B cell-based protein secretion6). This result demonstrates (1) that PVCs are functional in circulation (building on our 2023 manuscript, which only showed in vivo activity via local stereotaxic injection in the brain); and (2) that in vitro reprogrammed PVCs retain their desired specificity after introduction into animals (confirming the result from Fig. 2j). This result also raises the possibility of using PVCs for therapeutic interventions targeting B cells.

3.In the landscape of viral and nonviral delivery systems, how can readers frame and understand SPEAR? There are many highly efficient ways to transfect cells, and it was unclear if, or how, SPEAR can be leveraged to do things that current approaches cannot already do. If the advance centers only on creativity and engineering, and not translational science, this can be stated as well.

In general, the focus of this work was on establishing novel engineering methods (rather than translational applications) for PVCs; still, we are excited about the future translational possibilities for this novel delivery platform. Specifically, we believe the system offers the following advantages:

Modularity: Due to the unique structure of the PVC, targeting and cargo loading take place in structurally independent locations within the complex, such that both can be tuned independently of the other. In addition, we have now demonstrated two separate cargo loading mechanisms for this system (tube-based loading and spike-based loading), which can be used in tandem, allowing mixtures of defined cargos to be delivered simultaneously (Fig. 1g).

Cargo flexibility: We have now demonstrated that PVCs can be loaded with a wide variety of molecular cargos (proteins, RNPs, ssODNs, and even combinations of all three). This system does not appear limited by cargo size constraints (as with many other vectors, such as AAVs) and is not limited to only one category of cargo (protein, RNA, or DNA) as with many other vectors. We are exploring the possibility of leveraging SPEAR to help internalize much larger cargos, including VLPs1, protein nanoparticles2, or DNA origami cages3, many of which lack native mechanisms for membrane transit or endosomal escape.

Delivery of folded cargos: Most existing protein delivery systems unfold cargos prior to delivery. However, because many proteins cannot spontaneously re-fold once inside the target cell, technologies capable of delivering folded proteins are highly sought-after. Notably, we have shown that SPEAR can translocate cargos directly through the cellular membrane without loss of native folding, allowing for single-step delivery of macromolecular assemblies (Cas9 RNPs).

Target specificity: We have shown that PVC activity is tightly regulated and requires specific recognition of a target receptor by the tail fiber (Pvc13). Notably, PVC activity is thought to require binding by multiple tail fibers simultaneously—in contrast to many other vectors, in which a single binding event is often sufficient to trigger internalization—resulting in an impressive level of target specificity for this system (see Fig. 3 from our 2023 paper for a more detailed analysis of PVC specificity).

Efficient production and functionalization: Because PVCs can be efficiently purified from bacteria, and can then be retargeted and loaded with cargos in vitro (Fig. 2 of this manuscript), we believe this system could be efficiently scaled up for downstream manufacturing.

Novel mechanism of action: Finally, we argue SPEAR makes a meaningful contribution to the delivery space because it offers a fundamentally novel mechanism of action that has not been exploited by other vector technologies. This mechanism—direct membrane puncture—does not rely on native pathways for internalization, cellular trafficking, or host transcription/translation (as with many other vectors, which largely rely on receptor-mediated endocytosis or membrane fusion for entry), thus making it an ideal candidate for use in cell types and tissues which are metabolically inactive or senescent (a longstanding challenge in delivery) or in completely different organisms which are otherwise difficult to access with current delivery tools (e.g. plants, single-celled parasites, or even prokaryotes). We believe the contribution of a new category of vectors based on membrane-puncturing contractile injection systems will offer ample opportunity for future work to optimize delivery efficiency and further expand the potential application space for this new platform.

In summary, while not necessarily competitive with some other vectors (such as LNPs) in raw delivery efficiency in its current form, we believe SPEAR offers a favorable combination of cargo flexibility, target specificity, and modularity/programmability which sets it apart from existing delivery technologies. Whereas other delivery vehicles, such as AAVs and LNPs, have benefitted from years of development and optimization, PVCs represent a fundamentally novel technology with a radically different mechanism of action. In this study, we established several powerful new methods to functionalize PVC cargo loading and cellular targeting; we are optimistic that future studies will be able to leverage these approaches to demonstrate new therapeutic applications for these tools and increase their efficiency in cells.

4.It is currently unclear, particularly with the brief communication format, what are the broader impacts and potential utility of SPEAR. I suggest adding text to detail broader impacts and comments on how SPEAR could be used therapeutically or otherwise find utility.

In our revised manuscript, we included additional text to guide the reader's interpretation of the broader impacts of SPEAR in the context of therapeutic delivery:
Lines 119-124: Added a discussion of the benefits of SPEAR and potential downstream applications for this novel technology.
Lines 106-107: After our summary of the novel in vivo data, we added a statement of the potential utility of SPEAR in gene therapies targeting B cells.
Lines 89-91: Added an expanded explanation of the utility of ST/SC-based in vitro retargeting.

References

1. Nooraei, S. et al. Virus-like particles: preparation, immunogenicity and their roles as nanovaccines and drug nanocarriers. J. Nanobiotechnology 19, 59 (2021).

2. Olshefsky, A., Richardson, C., Pun, S. H. & King, N. P. Engineering Self-Assembling Protein Nanoparticles for Therapeutic Delivery. Bioconjug. Chem. 33, 2018–2034 (2022).

3. Kumar, M., Jha, A. & Mishra, B. DNA-Based Nanostructured Platforms as Drug Delivery Systems. Chem Bio Eng. 1, 179–198 (2024).

4. Berkson, J. D. et al. Phage-specific immunity impairs efficacy of bacteriophage targeting Vancomycin Resistant Enterococcus in a murine model. Nat. Commun. 15, 2993 (2024).

5. Archana, A., Patel, P. S., Kumar, R. & Nath, G. Neutralizing antibody response against subcutaneously injected bacteriophages in rabbit model. VirusDisease 32, 38–45 (2021).

6. David, M. et al. Production of therapeutic levels of human FIX-R338L by engineered B cells using GMP-compatible medium. Mol. Ther. Methods Clin. Dev. 31, (2023).

7. Jiang, F. et al. Cryo-EM Structure and Assembly of an Extracellular Contractile Injection System. Cell 177, 370-383.e15 (2019).

8. Kreitz, J. et al. Programmable protein delivery with a bacterial contractile injection system. Nature 616, 357–364 (2023).

9. Li, K., Wang, G., Andersen, T., Zhou, P. & Pu, W. T. Optimization of Genome Engineering Approaches with the CRISPR/Cas9 System. PLOS ONE 9, e105779 (2014).

10. Blencowe, H. et al. Rare single gene disorders: estimating baseline prevalence and outcomes worldwide. J. Community Genet. 9, 397–406 (2018).

Version 1:

Reviewer comments:

Reviewer #1

(Remarks to the Author)
The authors have adequately answered the comments from the first round of review. I have no additional comments.

Reviewer #2

(Remarks to the Author)
The authors have appropriately addressed our comments in the revised submission. Thank you.

Reviewer #3

(Remarks to the Author)
My comments were largely addressed, and I support the manuscript for publication.

Open Access This Peer Review File is licensed under a Creative Commons Attribution 4.0 International License, which permits use, sharing, adaptation, distribution and reproduction in any medium or format, as long as you give appropriate credit to the original author(s) and the source, provide a link to the Creative Commons license, and indicate if changes were made.
In cases where reviewers are anonymous, credit should be given to 'Anonymous Referee' and the source.
The images or other third party material in this Peer Review File are included in the article's Creative Commons license, unless indicated otherwise in a credit line to the material. If material is not included in the article's Creative Commons license and your intended use is not permitted by statutory regulation or exceeds the permitted use, you will need to obtain permission directly from the copyright holder.
To view a copy of this license, visit https://creativecommons.org/licenses/by/4.0/
